# Effects of Different Tissue Microenvironments on Gene Expression in Breast Cancer Cells

**DOI:** 10.1371/journal.pone.0101160

**Published:** 2014-07-08

**Authors:** Gaelle Rondeau, Parisa Abedinpour, Prerak Desai, Veronique T. Baron, Per Borgstrom, John Welsh

**Affiliations:** Vaccine Research Institute of San Diego, San Diego, California, United States of America; West Virginia University, United States of America

## Abstract

In metastasis, circulating tumor cells penetrate the walls of blood vessels and enter the metastatic target tissue, thereby becoming exposed to novel and relatively unsupportive microenvironments. In the new microenvironments, the tumor cells often remain in a dormant state indefinitely and must adapt before they are able to successfully colonize the tissue. Very little is known about this adaptive process. We studied temporal changes in gene expression when breast cancer cells adapt to survive and grow on brain, bone marrow, and lung tissue maintained in an *in vivo* culture system, as models of the metastatic colonization of these tissues. We observed the transient activation of genes typically associated with homeostasis and stress during the initial stages of adaptation, followed by the activation of genes that mediate more advanced functions, such as elaboration of cell morphology and cell division, as the cells adapted to thrive in the host tissue microenvironment. We also observed the temporary induction of genes characteristic of the host tissue, which was particularly evident when tumor cells were grown on brain tissue. These early transient gene expression events suggest potential points of therapeutic intervention that are not evident in data from well-established tumors.

## Introduction

Signals supplied by the local microenvironment control gene expression during development [1] and when cells are placed in novel tissue microenvironments for investigative purposes [2], [3]. Similarly, cancer cells in primary tumors respond to signals present in their local microenvironment [4] and reciprocally alter gene expression in the local stroma [5]. Similar reciprocal effects can be expected between metastatic tumors and their target tissue microenvironments. The individual steps in the progression of primary cancer to metastatic cancer have been reviewed [6], [7], and there are extensive gene and protein expression data from preinvasive and invasive primary tumors, metastatic tumors, and circulating tumor cells. The transitions between these physiological compartments, i.e. crossing the basement membrane in the epithelial-to-mesenchymal transition (EMT), entering the circulatory system, extravasating into and colonizing a target tissue, have all been studied using diverse strategies [7].

A common approach used to study extravasation and colonization of target tissue has been comparison of cells serially transplanted to select for those able to invade a specific tissue or organ with different efficiencies. For example, MDA-MB-231 cell lines have been selected to efficiently metastasize to the brain [8]. Gene expression analysis of cells selected in this manner was used to identify genes that mediate breast cancer metastasis to the brain [9]. Similarly, gene expression in 4T1 cells, which metastasize to bone, lung, and lymph nodes, was compared to gene expression in 4TO7 cells, which were derived from the same primary tumor, and which extravasate into but fail to colonize the lung [10], to identify genes that mediate colonization of lung [11]. In those experiments, cells introduced into the circulation were cultured from the target tissue, and this process was repeated, resulting in cell lines with progressively more aggressive ability to extravasate, survive, and colonize the target tissue. However, the efficiency of colonization depends on post-extravasation cell death and on the duration of post-extravasation dormancy [12]. Consequently, correlations between gene expression and metastatic potential for cell lines selected for differential metastatic potential by this approach do not resolve the individual contributions of extravasation, survival, and colonization. Each of these steps in metastasis engages distinct cell-physiological processes and represents conceptually distinct points of potential therapeutic intervention. In addition, once a cell has extravasated and learned to survive, the rate of colonization can increase progressively, and mechanisms underlying the acquisition of more aggressive growth have not been described. Events that ensure the survival of the cell immediately after extravasation are not understood.

We explored the time-dependence of differential gene expression when cancer cells were directly embedded in different tissue microenvironments, as a model for the immediate events that occur during post-extravasation colonization in different metastasis microenvironments. We observed early responses which include the up-regulation of genes normally involved in material homeostasis, whereas later changes include the up-regulation of genes normally involved in cell shape, division and motility, in accordance with gene expression data on established tumors. Of particular interest, we observed that breast cancer cells initially express genes that are characteristic of the surrounding tissue. This was most evident when breast cancer cells were co-cultivated with brain tissue, for which a large number of tissue specific genes can be identified, but may also occur when the co-cultivated tissue is bone marrow or lung. Many of the changes in gene expression induced by co-cultivated tissues and observed in early passages did not persist in later passages. Such transient gene expression must also occur in natural metastasis and cannot be discovered by examining gene expression in natural primary tumors or established natural metastatic tumors, inasmuch as they may precede tumor growth. Therefore, therapeutic targets to delay or prevent the further development of early metastatic lesions might be identified by exploring early gene expression events when tumor cells first encounter a foreign tissue environment.

## Results

The processes by which disseminated tumor cells (DTC) adapt to different tissue microenvironments are poorly understood. Typically, disseminated tumor cells experience a dormant phase immediately after extravasation that may persist for many years. These disseminated tumor cells reside in foreign tissue microenvironments as single cells until they acquire the ability to divide. We designed an experiment to identify genes that are differentially regulated during this adaptive period in brain, bone marrow, and lung tissue. We circumvented the extravasation step, itself, by directly grafting tumor cell spheroids onto these tissues prepared using a dorsal skinfold chamber *in vivo* co-culture system. The dorsal skinfold chamber is a small stainless steel and glass device that is surgically attached to a fold in the skin on the back of a mouse, in such a way as reveal the underside (i.e. the hypodermis) of the skin on one side of the fold through hole in the other side, which is protected by a microscopy window. Minced tissues grafted to the hypodermis in dorsal skinfold chambers survive and revascularize, and retain their original character with respect to several molecular markers [13]. Tumor cells can be grown on these tissue substrates by grafting tumor cell spheroids, yielding a pseudo-orthotopic intravital microscopy model for tumor growth in which growth rate and other parameters can be monitored [14]. The tumor cells can be recovered from the dorsal skinfold chambers and analyzed for gene expression.

### Tumor cell adaptation to different tissues

**Figure 1:**
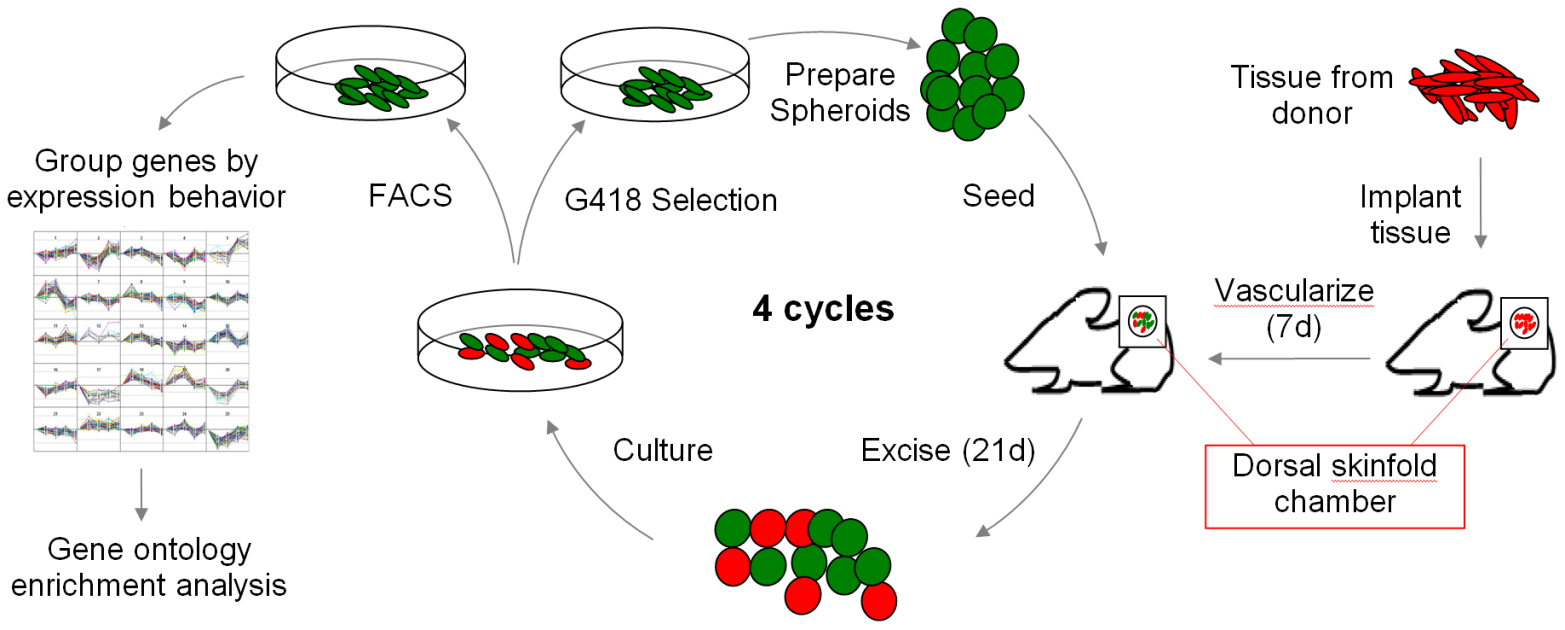
Outline of the experiment. Tissue (brain, bone marrow, or lung) is taken from a donor mouse, minced with a razor, and placed in a dorsal skinfold chamber. After ∼7 days, when the tissue has developed vasculature, tumor spheroids prepared initially from the parental (N202) cell line were grafted on top of the vasculaturized tissue bed. After 21 days, the tumor and tissue were placed in cell culture, allowing tumor cells and cells from the tissue to grow. These were selected using G418 and placed back in tissue culture to increase their numbers. These cells were converted into spheroids and reintroduced onto vascularized tissue in a new dorsal skinfold chamber or sorted on the basis fo GFP fluorescence and analyzed for differential gene expression. This was repeated for up to 4 cycles (P0-P4). Genes were grouped by k-means according to differential gene expression in P1-P4 relative to the parental cell line (P0). These k-means groups were then examined for enrichment of Gene Ontology Biological Process Terms.

**Fig.1** presents an outline of the experimental design used in the present study. We prepared brain, bone marrow, and lung tissues in this manner and grafted the murine Her-2/neu-positive transgenic mouse breast cancer cell line, N202, transformed to express H2B-GFP, as described previously [15]. Three weeks after *in vivo* seeding, cells from the resulting N202 tumors were grown *in vitro*, in the presence of G418, which selected for the presence of the H2B-GFP cassette. Spheroids were prepared from these cells and were reintroduced into the *in vivo* culture system for about three weeks. This process was repeated for up to four cycles. Cells grown in this manner on brain tissue for four cycles initially grew more slowly *in vitro* than did the parental cell line, but eventually returned to or surpassed the initial growth rate. Growth *in vitro* and *in vivo* correlated well (**Figs. S1A, D, G**). In bone marrow, a steady increase in growth rate was observed both *in vitro* and *in vivo* (**Figs. S1B, E, H**), also with strong correlation. Although strong correlation between *in vitro* and *in vivo* growth was not anticipated, it supports the conclusion that some characteristics acquired during *in vivo* adaptation persist during *in vitro* growth. Adaptation to lung tissue was irregular and correlation between *in vivo* and *in vitro* growth was poor. The lack of correlation between growth *in vivo* vs. *in vitro* for lung tissue (**Figs. S1C, F, I**), appeared to be due to growth arrest caused by hemolysis in the IVM chamber at several time points (not shown). Increases in tumor growth rates with serial transplantation have been observed previously, and are generally understood to reflect adaptation to the microenvironment [16], [17].

**Figure 2:**
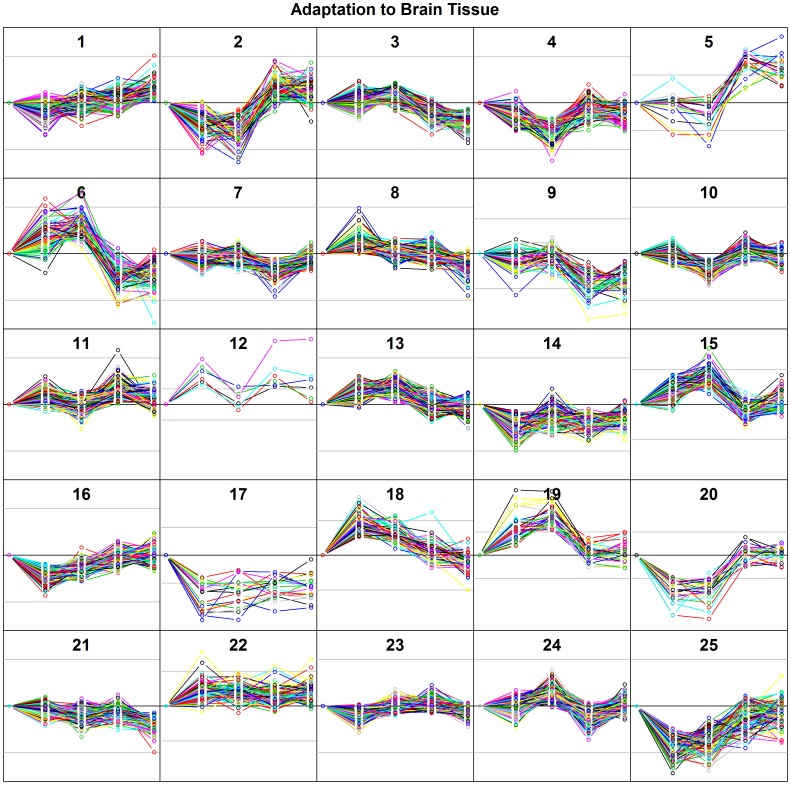
K-means grouping of gene expression fold changes during adaptation of N202 cells to brain tissue. Genes were grouped according to expression profiles over time using k-means and assuming 25 groups (i.e. N = 25). The central black line in each frame represents no change, i.e. 1-fold changes, and the gray lines represent 4-fold increases or decreases, relative to the parental cell line. Horizontally, the five positions along the x-axis represent sequential *in vivo* passages, P0 (i.e. parental cells), P1, P2, P3, P4, and gene expression analysis was performed on cells expanded *in vitro* from each of these passages. We then explored how GO Biological Process Terms distributed among these 25 groups.

Cancer cells subjected to selection for growth in a foreign tissue microenvironment exhibit changes in gene expression some of which persist when the cells are removed from the foreign tissue microenvironment and grown *in vitro*. Gene expression profiles for parental cells (P0) and passages 1-4 (P1-P4) were generated from the *in vitro* cell cultures after cell sorting. Genes were assigned to groups by k-means, based on their expression trajectories, which varied as the cells adapted to the brain, bone marrow, and lung tissue microenvironments over serial passages. **Fig. 2** shows graphs of k-means groups for cells grown on brain tissue at passages P0-4 assuming 25 k-means groups. These k-means groups reflect the coordinated regulation of the member genes over successive passages. The extent to which these changes reflect *bona fide* genetic selection versus epigenetic alterations is unknown, but either mechanism allows for selection of cells in which gene expression promotes or is at least neutral relative to survival and cell proliferation, which form the basis of selection. The corresponding graphs for bone marrow and lung can be found in **Figs. S2B and C**. The trajectories of individual genes and other sorting tools can be found at http://www.voxvill.org.

In this discussion, k-means groups for brain, bone marrow and lung are designated “BN_N_ X”, “BM_N_ X”, and “LN_N_ X”, where the subscript N refers to the number of k-means groups and X refers to a specific k-means group. There is no rigorous method to define the correct number of groups (see Materials and Methods). For most of the discussion that follows, N = 25 was chosen subjectively to resolve between clearly distinct temporal patterns, but to avoid separating similar temporal patterns on the basis of noise. Other choices for N can be explored at http://www.voxvill.org. GO Biological Process Terms (www.geneontology.org) associated with the genes in each k-means group at statistically significant frequencies were determined and are italicized in the text that follows. GO Biological Process Terms relevant to the discussion and the corresponding GO IDs are listed in **Table 1**, with p-values from Fisher's exact test [18] corrected to indicate false discovery rates (q-values) [19]. Several physiological and developmental functions commonly associated with the development of tumors, including homeostasis, cell cycle, motility and morphology, angiogenesis, and functions that reflect perturbations in protein dynamics and innate immunity, are apparent in these data and are listed in this table. Following a brief discussion of the induction of these various functions, we show that cancer cells grafted onto brain tissue initially respond to this microenvironment by temporarily elevating the expression of genes normally associated with neurobiological functions. Other significant GO terms, of which there are many, can be explored in detail at http://www.voxvill.org. The following discussion pertains to GO terms that were significant with qval ≤0.05.

**Table 1:** Significant GO Biological Process Terms Illustrating Sequential Adaptation of N202 Cells to Brain Tissue.

Function	KMG	GO ID	GO Biological Process Term	qval*
Homeostasis	BN_25_ 6	0006814	sodium ion transport	0.0006
		0015807	L-amino acid transport	0.0008
		0006811	ion transport	0.0009
		0003333	amino acid transmembrane transport	0.006
	BN_25_ 15	0006811	ion transport	0.008
		0006820	anion transport	0.03
		0015991	ATP hydrolysis coupled proton transport	0.004
	BN_25_ 18	0055072	iron ion homeostasis	0.0009
		0015809	arginine transport	0.042
	BN_25_ 19	0015837	amine transport	0.018
(many others: See Table S1)				
Cell cycle, Locomotion	BN_25_ 2	0032321	positive regulation of Rho GTPase activity	0.001
And Morphology		0030036	actin cytoskeletal organization	0.003
		0032956	regulation of actin cytoskeleton organization	0.016
		0051017	actin filament bundle assembly	0.019
	BN_25_ 4	0007049	cell cycle	7×10^−11^
		0051301	cell division	4×10^−7^
		0007067	mitosis	2×10^−6^
		0006260	DNA replication	8×10^−6^
		0007076	mitotic chromosome condensation	4×10^−7^
		0006281	DNA repair	0.0002
		0030261	chromosome condensation	0.0004
		0007059	chromosome segregation	9.7×10^−4^
		0006334	nucleosome assembly	0.001
		0006487	protein N-linked glycosylation	0.002
		0006284	base-excision repair	0.006
		0007099	centriole replication	0.009
Angiogenesis	BN_25_ 15	0001569	patterning of blood vessels	0.004
		0001944	vascular development	0.004
	BN_25_ 17	0045747	positive regulation of Notch signaling pathway	0.0005
	BN_25_ 20	0001525	angiogenesis	0.003
	BN_25_ 25	0016525	negative regulation of angiogenesis	0.03
Endoplasmic Reticulum,	BN_25_ 15	0034976	response to endoplasmic reticulum stress	0.001
Stress And Innate Immunity		0030968	endoplasmic reticulum unfolded protein response	0.006
		0032436	positive regulation of proteasomal ubiquitin-dependent protein catabolic process	0.01
	BN_25_ 19	0008228	opsonization	0.0003
		0006935	chemotaxis	0.0004
		0043032	positive regulation of macrophage activation	0.0007
		0050766	positive regulation of phagocytosis	0.002

Several functions important in tumor development are prominently represented in genes grouped by their expression behavior over time. The group designation (KMG) references the groups in Fig. 2, such that the relative timing of the induction or repression of these functions can be seen.

### Functional evolution during adaptation

Material Homeostasis The molecular determinants present in a metastatic tumor microenvironment are generally thought to be initially inhospitable to the extravasated disseminated tumor cells, possibly leading to dormancy. Extravasation necessarily implies the immediate proximity of microvasculature, therefore it cannot be taken for granted that extravasated disseminated tumor cells have limited access to nutrients due to a delay in the activation of neovascularization programs. In our experimental model, tumor spheroids consist of only a few tens of thousands of cells grafted onto a fully vascularized bed of tissue, suggesting that diffusion is unlikely to be limiting. Nevertheless, material balance, particularly ion balance, appears to be a key variable. Several k-means groups (**Fig. 2**) consist of genes that are up-regulated in P1 and P2. The genes in these groups manage early responses of the tumor cells to the novel tissue microenvironment and are often involved in ion transport. To explore this, we determined the GO Biological Process Terms that were disproportionately represented by genes in each k-means group. In these early response groups, the most significant GO terms (qval≤0.05) in **BN_25_ 6** (**Fig. 2** **and** **Table 1**) are *sodium ion transport*, *L-amino acid transport*, and *ion transport*. *Ion transport*, *anion transport*, and *ATP hydrolysis coupled proton transport* are highly significant in **BN_25_ 15**, *iron ion homeostasis* and *arginine transport* are significant in **BN_25_ 18**, and *amine transport* is significant in **BN_25_ 19**. All of these groups consist of genes that are sharply up-regulated in P1 or P2. The GO annotations of statistical significance associated with these groups can be found in **Table S1**, along with a large collection of additional genes characterized by up-regulation in P1 and associated GO Biological Process Terms relating to transport that are statistically significant with qval≤0.05. These results indicate that the cells first respond to this novel brain tissue microenvironment by regulating genes involved in transport of materials to maintain homeostasis.

The immediate importance of ion homeostasis in adaptation to bone marrow can be seen most easily in the increase in expression of genes in **BM_25_4** in which *anion transport*, *sodium ion export, cellular cation homeostasis, elevation of cytosolic calcium ion concentration*, *positive regulation of potassium ion transport, sodium ion transmembrane transport*, and others. In general, adaptation to this microenvironment appears to be less demanding with regard to ion homeostasis, displaying 36 transport-related GO Biological Process Terms compared to 68 such terms for the experiment using brain tissue.

In the lung tissue experiment, there are 23 such terms related to initial up-regulation of gene expression. Although the *in vitro* growth rate for cells grown on lung tissue did not correlate well with *in vivo* growth, several of the k-means groups indicate a general, if not smooth, adaptive response to the lung microenvironment, insofar as genes in groups **LN_25_2, 7, 8, 20, 23**, and **25** undergo net increases over adaptation time, while genes in groups **LN_25_1, 10, 16**, and **LN_25_22** undergo net decreases. Early responses involving ion transport in lung include *iron ion transport* in **LN_25_8**, *transmembrane transport and sodium ion transport* in **LN_25_12**, and *ion transport* in **LN_25_23** are up-regulated. These observations suggest that the brain tissue microenvironment presents a greater adaptive challenge to ion homeostasis than bone marrow or lung tissue, but that all three respond significantly by activating functions concerned with homeostasis.

Although adaptive response to ion imbalance is an obvious possible explanation for induction of these functions, another possibility is that their induction in the brain tissue experiment is due to a local microenvironment that vigorously controls the expression of genes involved in maintenance of the blood-brain barrier, which tightly regulates ion flow. Consistent with this interpretation is ∼7-fold up-regulation of Jam2, ∼4-fold up-regulation of Cldn1, ∼2-fold up-regulation of Cldn3, Cldn12, and Magi2, which are all tight junction components [20], signifying a response to the microenvironment that is not ostensibly homeostatic in nature. Inappropriate regulation of ion flow caused by defective or incomplete microenvironmental regulation of ion channels may actually increase rather than decrease stress, suggesting that only those cells that evolve to ignore these microenvironmental signals may be able to proceed.

#### Cell growth, morphology and locomotion

In natural disseminated tumor cell dormancy, the tumor cells fail to grow when they first encounter a foreign tissue microenvironment. We observed parallel behavior when N202 cells were transplanted onto brain tissue. The cell-physiological basis for this growth arrest is not well-understood, but a generic response to stress may be responsible. Alternatively, genes exist whose primary selected functions may include prevention of metastasis [21]. Gene expression in a third of the k-means groups (**BN_25_ 2, 4, 5, 16, 20, and 25**) is repressed during P1 and P2, during which time cell growth is slow and cell morphology is relatively simple, whereas expression in other groups (**BN_25_ 6, 13, 15, 18, 19, and 24**) is elevated during this initial phase and anti-correlated with growth rate and morphological complexity (**Figs. S1 and S3**). **BN_25_ 4**, which reaches its lowest point at P2 when cell cycle is at its slowest, contains significant GO terms most directly related to cell cycle (**Table 1**). The genes annotated with the GO terms discussed in this and the next section are listed in **Table S2**. During adaptation, cell shapes became less complex in P1 and P2, and then became more complex in P3 and P4 (**Fig. S3**), consistent with the regulation of cytoskeletal processes by Rho GTPases [22]. These processes are enriched in **BN_25_ 2** and **BN_25_ 4**, coincident with the timing of changes in proliferation and morphological complexity (**Table 1**). >32-fold up-regulation places the Kit proto-oncogene from **BN_25_ 19** among the most highly up-regulated genes observed in these experiments, although here, the expression of Kit proto-oncogene is inversely correlated with rapid growth.

In the bone marrow experiment, *cell-matrix adhesion* and *positive regulation of monocyte chemotaxis* are significant in **BM_25_ 4**, in which increases in gene expression correlate well with cell growth rate, as does **BM_25_ 16**, which comprises genes associated with GO terms concerning proliferation and mobility including *positive regulation of cell proliferation* and *positive regulation of cell motility*. Although these correlations are quite evident and statistically significant in the experiment using bone marrow, both bone marrow and lung tissue appear to be able to immediately support growth of the parental N202 cells with minimal initial adaptation. In lung, growth does not trend steadily higher toward the end of the 4 adaptation cycles, and although many genes in **LN_25_ 14** are associated with the term *mitosis, cell cycle* and *cell division*, the first of which achieves the highest significance level of any term in the experiment with lung tissue, this k-means group is distinguished in that is among the most weakly correlated with changes in growth, suggesting that genes associated with the mitotic machinery were left unaffected during this adaptation series. *Regulation of cell migration* is significant in **LN_25_ 23**, in which gene expression increases, overall, during adaptation.

#### Innate immunity and endoplasmic reticulum stress

Improper regulation of Ca^2+^ and other forms of homeostasis perturbations lead to endoplasmic reticulum stress [23], and there is some indication that the unfolded protein response and innate immunity share regulatory genes [24]. Genes in **BN_25_ 15 and 19** reach their maxima at P2, and are enriched in terms related to endoplasmic reticulum stress and innate immune functions, respectively. In **BN_25_ 15**, genes that are induced in P1 and P2 and return to control levels by P3 are enriched in the terms *response to endoplasmic reticulum stress*, *endoplasmic reticulum unfolded protein response*, and *positive regulation of proteasomal ubiquitin-dependent protein catabolic process*. Concordantly, anti-apoptotic Bcl2, which is found in **BN_25_ 6** and protects against endoplasmic reticulum stress [25], is at its highest level in P2, and has been associated with B-cell lymphomas and other cancers [26].

Several genes initially up-regulated in P1 and P2 in group **BN_25_ 19** are associated with innate immune system functions such as *opsonization*, *chemotaxis*, *positive regulation of macrophage activation*, *positive regulation of phagocytosis* and others. In clinical tumor studies of gene expression, genes related to innate immunity are difficult to interpret correctly due to the infusion of macrophage and other circulating cell types, also observed in nude mouse animal models. In the present experiments, these gene expression measurements are performed on pure cultures of tumor cells sorted on the basis of GFP fluorescence, and therefore cannot reflect contamination by non-tumor cell types. Therefore, these innate immune responses are genuine tumor responses. *Inflammatory response* and *immune response* correlate with rapid growth *in vitro* in cells adapted to grow on lung tissue in **LN_25_ 23**.

#### Angiogenesis

Angiogenesis promotes tumor survival and growth, and in this pseudo-orthotopic model, neovasculature arises from pre-existing vasculature in the local stromal tissue [14]. **BN_25_ 20** contains genes that are sharply *down-regulated* during P1 and P2, and then rebound in P3 and P4. Several of the genes in this group, Plau, Col8a1, and Nrp1, are associated with *angiogenesis* and Ddah1 promotes endothelial cell proliferation [27]. In **BN_25_ 25**, Adamts1 and Sema4a, associated with *negative regulation of angiogenesis*, are sharply down-regulated. None of these genes is similarly regulated in the bone marrow or lung tissue microenvironments. In **BN_25_ 17**, Eya1 and Hey1, regulate sprouting angiogenesis [28], are associated with *positive regulation of Notch signaling pathway*, and are strongly down-regulated in all three tissues. Nevertheless, revascularization in all three tissues is robust (**Figs. S4A,B**), possibly indicating that revascularization may be driven by the three tissue microenvironments, rather than by the tumor cells. The tumor cells adapting to brain tissue do provide several pro-angiogenic functions temporarily, e.g. in **BN_25_ 15**, Plxnd1, Vangl2, Fzd2, Fzd5, and Vegfa promote *patterning of blood vessels* and *vascular development*. In **LN_25_ 20**, *positive regulation of angiogenesis* is a later event that correlates with more rapid growth.

The foregoing discussion in this and in previous sections indicates that tumor cells growing in the pseudo-orthotopic skinfold chamber model modulate gene expression in ways that make sense within the context of our understanding of cancer. The tumor cells must adjust to the immediate stresses of the foreign tissue microenvironment for survival. During this time, external signals received by the cells and cell-autonomous programs adjust to achieve compatibility vis-à-vis appropriate protein synthesis and trafficking. Inadequacies of the local microenvironment with respect to material exchange lead to promotion of angiogenesis. More complex cell physiology such as changes in cell growth and morphology occur only after these more basic problems are solved. These challenges to tumor cell growth appear to be more severe in brain tissue than in bone marrow or lung tissue.

### Host tissue genes induced in tumor cells

We infer from these analyses that breast cancer-derived N202 cells transplanted on brain tissue also initially express genes characteristic of the brain tissue microenvironment. For this analysis, we defined GO Neurobiological Process Terms as GO Biological Process Terms containing one or more of the character strings ‘axon’, ‘neuro’, ‘neural’, ‘brain’, ‘neocortex’, ‘nerv’, ‘glial’, ‘hippocampal’, ‘hippocampus’, ‘cerebellar’, ‘cerebral’, ‘dopa’, ‘synaptic’, ‘sensory’, ‘astrocyte’, ‘olfactory’, and ‘memory’. **Table 2** lists significant (qval≤0.05) GO Neurobiological Process Terms from k-means groups in which genes were initially up-regulated in P1 by at least 1.5-fold, using N = 25. In which 34 genes associated with significant GNBP were up-regulated in P1, and 22 were down-regulated. Using various assumptions for N (i.e. N = 9, 16, 25, and 36), 71 genes associated with significant GO Neurobiological Process Terms were up-regulated in brain tissue by at least 1.5-fold in P1, compared with 14 and 20 in P1 in bone marrow and lung, respectively (**Table 3**). Occasionally genes annotated with GO Neurobiological Process Terms were assigned to groups in which no enrichment of GO Neurobiological Process Terms was observed. Independently of k-means analysis, enrichment of GO Neurobiological Process Terms in genes up-regulated in P1 by ≥1.5-fold was observed in the brain tissue experiment relative to non-GO Neurobiological Process Terms (p = 2×10^−5^, Fisher's exact test) and in P2 (p = 2×10^−3^, Fisher's exact test), and in the lung tissue experiment in P3 (p = 0.02, Fisher's exact test). These results indicate strong statistical support for the statement that brain tissue induces GO Neurobiological Process Terms in breast cancer cells in P1 and P2, that is, when the cells first encounter brain tissue. Some enrichment for GO Neurobiological Process Terms also occurs in lung, possibly related to lung innervation.

**Table 2:** Induction of neurobiological functions in N202 cells grafted onto brain tissue.

GO number	GO_Biological Process Term	KMG	+/-	Genes
0048846 0007409 0021972 0033563 0090260 0021836	axon extension involved in axon guidance, axonogenesis, corticospinal neuron axon guidance through spinal cord, dorsal/ventral axon guidance, negative regulation of retinal ganglion cell axon guidance, chemorepulsion involved in postnatal olfactory bulb interneuron migration	6	+	Slit2
0051402	neuron apoptotic process	6	+	Casp3
0046928	regulation of neurotransmitter secretion	6	+	Celsr1
0007422	peripheral nervous system development	6	+	Slc5a3
0001941	postsynaptic membrane organization	6	+	Chrnb1
0007411 0097156 0097155 0048681 0007399 0008347 0048710	axon guidance, fasciculation of motor neuron axon, fasciculation of sensory neuron axon, negative regulation of axon regeneration, nervous system development, glial cell migration, regulation of astrocyte differentiation	9	+	Epha4
0035633 0032227 0031915 0051968 0019233 0007613	maintenance of blood-brain barrier, negative regulation of synaptic transmission, dopaminergic, positive regulation of synaptic plasticity, positive regulation of synaptic transmission, glutamatergic, sensory perception of pain, memory	12	+	Ptgs2
0008090	retrograde axon cargo transport	13	+	Dst
0050905	neuromuscular process	13	+	Agtpbp1
0090177 0090179	establishment of planar polarity involved in neural tube closure, planar cell polarity pathway involved in neural tube closure	15	+	Vangl2
0090179	planar cell polarity pathway involved in neural tube closure	15	+	Fzd2
0032902	nerve growth factor production	15	+	Pcsk6
0033603	positive regulation of dopamine secretion	15	+	Slc18a1
0050953	sensory perception of light stimulus	15	+	Myo7a
0043382	positive regulation of memory T cell differentiation	15	+	Il23a
0021952 0021953 0001764 0042416 0042053	central nervous system projection neuron axonogenesis, central nervous system neuron differentiation, neuron migration, dopamine biosynthetic process, regulation of dopamine metabolic process	18	+	Nr4a2
0021952 0001764	central nervous system projection neuron axonogenesis, neuron migration	18	+	Dclk1
0014043 2000173 0060161 0022028	negative regulation of neuron maturation, negative regulation of branching morphogenesis of a nerve, positive regulation of dopamine receptor signaling pathway, tangential migration from the subventricular zone to the olfactory bulb	18	+	Lrrk2
0042135 0042420	neurotransmitter catabolic process, dopamine catabolic process	18	+	Maoa
0010975	regulation of neuron projection development	18	+	Klk6
0051971 0050966	positive regulation of transmission of nerve impulse, detection of mechanical stimulus involved in sensory perception of pain	18	+	Itga2
0007271	synaptic transmission, cholinergic	18	+	Chrm3
0008088 0002175	axon cargo transport, protein localization to paranode region of axon	19	+	Ugt8a
0006836	neurotransmitter transport	19	+	Slc6a14
0048170	positive regulation of long-term neuronal synaptic plasticity	19	+	Kit
0048676	axon extension involved in development	22	+	Mtap1b
2000734	negative regulation of glial cell-derived neurotrophic factor receptor signaling pathway involved in ureteric bud formation	22	+	Gata3
0043524 0048711	negative regulation of neuron apoptotic process, positive regulation of astrocyte differentiation	22	+	Clcf1
0043524	negative regulation of neuron apoptotic process	22	+	Gclm, Cln3
0031175	neuron projection development	22	+	Btg2
0031175 0010976	neuron projection development, positive regulation of neuron projection development	22	+	Cd24a
0010976 0048711	positive regulation of neuron projection development, positive regulation of astrocyte differentiation	22	+	Lif
0051930	regulation of sensory perception of pain	22	+	Edn1
0048484	enteric nervous system development	2	-	Phactr4
0001774	microglial cell activation	2	-	Jun
0002282	microglial cell activation involved in immune response	2	-	Tlr2
0021766	hippocampus development	2	-	Hdac1
0050772	positive regulation of axonogenesis	4	-	Sema4d, Shox2
0007411 0007638	axon guidance, mechanosensory behavior	9	-	Etv1
0007411 0007399	axon guidance, nervous system development	9	-	Sema5a
0007399	nervous system development	9	-	Sema7a
0007420	brain development	9	-	Sepp1
0048011	nerve growth factor receptor signaling pathway	9	-	Sort1
0048678	response to axon injury	14	-	Erbb2
0043525	positive regulation of neuron apoptotic process	16	-	Map3k11, Bcl2l11, Ptprf, Cdc42
0043526	Neuroprotection	17	-	Ccl5
0045664	regulation of neuron differentiation	17	-	Eya1
0001580	detection of chemical stimulus involved in sensory perception of bitter taste	17	-	Rtp4
0007413 0030517 0048843 0048841 0048485 0021636	axonal fasciculation, negative regulation of axon extension, negative regulation of axon extension involved in axon guidance, regulation of axon extension involved in axon guidance, sympathetic nervous system development, trigeminal nerve morphogenesis	20	-	Nrp1
0010977	negative regulation of neuron projection development	20	-	Lgals1
0070997	neuron death	20	-	Plau
0001956	positive regulation of neurotransmitter secretion	25	-	Sphk1

qval≤0.05, N = 25, P1 Ratio≥1.5-fold.

Genes typically associated with neurobiological functions tend to be induced more frequently than expected in groups defined by early increases in gene expression (qval≤0.05, N = 25, P1 Ratio≥1.5-fold). Genes with neurobiological functions were defined as those having GO Biological Process Terms containing partial matches with “axon”, “neuro”, “neural”, “brain”, “neocortex”, “nerve”, “glial”, “hippocampal”, “hippocampus”, “cerebellar”, “cerebral”, “dopa”, “synaptic”, “sensory”, “astrocyte”, “olfactory”, and “memory”.

**Table 3:** Genes associated with neurobiological GO processes* in the first passage (P1).

Expt	UP	DN	UP.cnt	DN.cnt
BM_9-36_	Edn1 Fam134b Hoxa1 Klk8 Ndrg1 Nr4a2 Palld Pdzrn3 Ptgs1 Ptgs2 Rac3 Sfrp1 Tlr2 Tubb2b	Bcl2 Casp3 Dll1 Egr1 Erbb2 Etv1 Etv5 Eya1 Hes1 Itga2 Sema5a Sema7a Slc5a3 Slit2 Sod1 Vim	14	16
BN_9-36_	Aars Agtpbp1 Areg Arhgef7 Atxn2 Btg2 Ccl2 Cd24a Cdk5 Celsr1 Chac1 Chrm3 Chrnb1 Clcf1 Cln3 Cobl Dbn1 Dclk1 Dst Edn1 Epha4 Etv1 F2r Flot1 Fzd2 Gata3 Gdf11 Gli3 Hif1a Hmga2 Hoxa1 Il23a Itga1 Itga2 Jhdm1d Kit Klk6 L1cam Lif Lmo4 Lrrk2 Maoa Mapk8 Mtap1b Myo7a Ndel1 Nr4a2 Pcsk6 Prmt1 Ptgs2 Ptk2b Rnd1 Rora Scrib Sema3b Sema3c Sema5a Sema7a Serpine2 Slc18a1 Slc1a2 Slc6a14 Slit2 Syngr1 Tbc1d24 Tcfap2a Tgfb2 Ugt8a Vangl2 Vegfa Wnt7b	Adam8 Arrb1 Bcl2l11 Casp6 Casp9 Ccl5 Cdc42 Erbb2 Etv1 Eya1 Hdac1 Hipk2 Jak2 Jun Lgals1 Lgmn Lrp8 Map3k11 Melk Ndrg1 Nrp1 Ntn1 Phactr4 Plau Ptprf Rb1 Rtp4 Sema4d Sema7a Shc1 Shox2 Six1 Sod1 Sort1 Sphk1 Tlr2 Utp11l	71	37
LN_9-36_	Agrn Arsa Arsb Cdon Chd7 Efna3 Eya1 Gpr115 Gpr56 Grhl3 Hoxa1 Lphn2 Maoa Nf1 Nr4a2 Pdzrn3 Ptgs2 Tcfap2a Tlr2 Trp53bp2	Ablim1 Adm Atp6v1b1 Casp3 Cav2 Clcf1 Crlf1 Dll1 Efnb1 Egln3 Egr1 Etv1 Gnas Hes1 Id4 Itga2 Klk6 Lynx1 Notch1 Nrtn Palm Plau Rarb Rtp4 Sema3e Sema5a Sema7a Shox2 Slit2	20	29

*Neurobiological GO processes were defined as those GO Biological Process Terms containing one of the following strings: ‘axon’, ‘neuro’, ‘neural’, ‘brain’, ‘neocortex’, ‘nerv’, ‘glial’, ‘hippocampal’, ‘hippocampus’, ‘cerebellar’, ‘cerebral’, ‘dopa’, ‘synaptic’, ‘sensory’, ‘astrocyte’, ‘olfactory’, and ‘memory’. The genes listed comprise the union of sets of genes associated with significant (i.e. qval≤0.05) neurobiological GO processes assuming 9, 16, 25, and 36 k-means groups. The column labeled UP lists those genes that were up-regulated with P1/P0 expression ratio≥1.5-fold. The column labeled DN lists those genes that were down-regulated with P1/P0 expression ratio≤1.5-fold. UP.cnt and DN.cnt contain the number of genes in UP and DN, respectively.

Some of the genes in **BN_25_ 6, 8, 18, and 19** are induced in P1 by ≥4-fold and perform neurobiological functions, but are not associated with enriched GO Neurobiological Process Terms annotations: Atp1b1, electrical excitability [29]; Chi3l1, neuroectoderm differentiation [30]; Chrm3, acetylcholine effects [31]; Prss12, synaptic function [32]; Slc38a1, glutamate transport [33]; Syne1, cerebellar ataxia [34]; Ugt8a, transfers galactose to ceramide [35]; Zc4h2, mental retardation [36]; Kctd12b, GABA(B) receptor subunit [37]; Lrrk2, Parkinson's disease [38]; Maoa modifies dopamine levels [39]; Neto2, glutamate signaling [40]; Nr4a2, schizophrenia [41]; Pcdh7, cell-cell interactions in the developing brain [42]; Slc1a1, post-synaptic glutamate neurotransmitter activity and schizophrenia [43]; and Slit2, brain vessel density and permeability [44].

Comparison of these differential expression data with data from human breast cancer metastases to the brain revealed strikingly high concordance. Among genes included in both this study and in the study by Palmieri et al. [45], 18/21 (86%) of the increases or decreases that accompany the transition from primary to metastatic cancer in clinical samples changed in the same direction in the transition from P0 to P2 in the present data. Permutation analysis over randomly selected groups of these 21 genes showed that this concordance in the direction of change was highly significant (p = 0.0002) (**Fig. S5**). Therefore, grafting of breast cancer cells onto brain tissue growing in a murine dorsal skinfold chamber recapitulates significant events that occur in human breast cancer brain metastasis.

These results indicate that the N202 cells initially interpret signals from the brain tissue microenvironment by expressing genes that are characteristic of the surrounding host tissue. The cells eventually evolve away from this orthotopic gene expression phenotype and toward a more rapid growth phenotype. In a biological replicate experiment, genes associated with neurobiological functions and induced in P1 and P2 in the first experiment (**Table 1**) were similarly induced in the replicate experiment by a ∼2∶1 margin (27∶13 induced vs. repressed in repP1 and 25∶15 induced vs. repressed in repP2). Among genes that were repressed in the first experiment, most were repressed in repP1 (5∶19, induced vs. repressed), but many of these returned to baseline or above by repP2 (15∶9, induced vs. repressed), similar to the later stage, P3 in the first experiment. Genes from **BN_25_ 5** in the replicate experiment were induced immediately, rather than exhibiting the two-cycle delay of the previous experiment. Modeling based on least-squares indicated that repP1 and repP2 corresponded most closely with times P2.60 and P3.68 in the original time series, indicating more rapid adaptation in the second experiment (**Fig. S6**). Such variation in timing would be expected of adaptation driven by stochastic processes, and as in natural cancer, this comparison emphasizes the “case study” nature of each independent adaptive series.

This observation of orthotopic gene expression was facilitated by the cell sorting, which allowed us to identify tissue specific gene expression without interference from contaminating cells from the host tissue, and also by extensive tissue-specific gene expression characteristic of the brain. Functions associated with bone marrow are often characteristic of inflammation as well, and as such cannot be used as evidence of orthotopic gene expression in this model. Nevertheless, their absence would be surprising and, indeed, many genes expressed in bone marrow and associated with inflammation are expressed when N202 cells are placed in contact with bone marrow. For example, in **BM_25_1**, Tlr2, Ripk2, Myd88 are initially up-regulated and control inflammatory responses [46], such as *positive regulation of interleukin-6 production, MyD88-dependent toll-like receptor signaling pathway*, and *positive regulation of tumor necrosis factor production* (see http://www.voxvill.org). *Immune response* due to Csf3, Cxcl5, and Cxcl1, and *inflammatory response* due to Itgb6, Cxcl5, and Cxcl1, are associated with P1 of **BM_36_25**. Several genes that change in P1 have functions in bone unrelated to inflammation, per se: in **BM_25_1**, Adamts1 is important in inflammation, but also in normal bone physiology [47] and metastasis to bone [48]; Alp1 is a tissue-specific alkaline phosphatase expressed in bone, liver, and kidney [49]; Nr4a2 can be induced in bone-marrow-derived mesenchymal stem cells [50]; Bcl9 is up-regulated in osteoarthritis [51]; Sfrp1, and Tob1 and Srfp1 are significantly associated with GO terms *bone trabecula formation* and *negative regulation of osteoblast differentiation*, respectively; Stc2 controls bone growth [52]; and Loxl4 is up-regulated in bone marrow but not brain and lung, and is involved in crosslinking collagen. In **BM_36_22**, in which genes are up-regulated only in P1, *hemopoietic progenitor cell differentiation* is significant due to Fst and Sfrp1, as are *negative regulation of bone remodeling*, *negative regulation of osteoclast differentiation*, *convergent extension involved in somitogenesis* and *bone trabecula formation*. In lung, *sterol biosynthetic process* achieve very high significance (qval = 4×10^−5^) in **LN_25_ 12**, an expression profile that tracks closely with *in vitro* growth.

These data show that cancer cells exposed to foreign tissue microenvironments initially respond by expressing genes typical of the cells that comprise the microenvironment. Genes in this early response class represent potential therapeutic targets to delay or prevent brain metastasis using drugs already in use for other purposes, including Alpl, Arg2, Bcl2, C3, Chrm3, Kit, Maoa, and Odc1 (see http://www.voxvill.org), with the essential caveat that the contributions of these genes to the fitness of the metastatic tumor are unknown. A few genes that can be inhibited by known drugs or chemicals are up-regulated at early times and persist or are up-regulated at later times, including Alpl, Plat, and Pnp. At least three drugable genes, Hdac1, Jun, and Vdr, are initially down-regulated by the brain tissue microenvironment, but then return to control levels when more rapid growth is observed, suggesting a delay in the timing of the potential therapeutic window. Interestingly, vorinostat, which inhibits Hdac1, also inhibits brain metastatic colonization in a breast cancer model [53]. In addition to genes that are induced early during adaptation, others induced in brain tissue, bone marrow and lung reach their highest levels in later passages, and these genes may also be of interest vis-à-vis drug targeting, insofar as blocking their later elevated expression may affect tumor cell fitness (see **Table S3**). Many other adaptive responses that occur in the serially transplanted tumor cells, including associations with stress and wound healing, innate immune responses, changes in metabolism and others, can be explored further at http://www.voxvill.org.

## Discussion

We allowed a murine breast cancer-derived cell line to adapt to various co-cultured tissues in an *in vivo* culture system, and measured gene expression over time as the cells adapted to the different tissues. We then grouped genes according to similarities in their temporal expression behavior by k-means and correlated the resulting groups with GO Biological Process terminology. This allowed us to describe a sequence of steps taken by a cancer-derived cell line during its adaptation to thrive in foreign tissue microenvironments. The sequential activation of functions relating, first, to homeostasis, then to management of endoplasmic reticulum stress, and finally to increased morphological complexity, migration, and cell division parallels the generally assumed model in which metastatic cells that have entered a novel tissue microenvironment by extravasation must first acquire the ability to survive before venturing to proliferate. Less attention has been paid to this early stage of metastatic colonization, in part because early metastatic tumors are microscopic and are therefore difficult to detect, and yield limited material for molecular analysis. In addition, contamination by the surrounding tissue can be difficult to exclude during direct molecular analysis, particularly in syngeneic models.

Breast cancer cells co-cultivated with brain tissue initially express a large number of genes typically expressed brain tissue, presumably due to microenvironmental signals emanating from the brain tissue, similar to stromal effects on gene expression during the epithelial-mesenchymal transition [54]. Induction of orthotopic gene expression was also observed when the co-cultivated tissue was bone marrow or lung tissue, although the observation of orthotopic gene expression with brain tissue was more dramatic, possibly due to a richer ontology vocabulary for brain versus bone marrow or lung. Our finding of remarkably high correspondence between gene expression in murine breast cancer cells adapted to grow on minced brain tissue cultivated *in vivo* and gene expression in clinical breast cancer metastases to the brain suggests that temporal changes observed in our model system are likely to recapitulate temporal phenomena in clinical disease.

We also observed that gene expression evolves during tumor cell adaptation to these various tissues, and that some early changes are temporary. This suggests that analysis of gene expression in well-established clinical tumors is unlikely to accurately reflect earlier gene expression events, some of which may represent novel clinical targets. In our experiments, 89 genes reached their maximum expression of ≥2-fold in tumor cells immediately upon co-cultivation with brain tissue, and there are drugs and small molecule inhibitors for 26 of these. This suggests potential strategies to prevent metastatic dissemination of breast cancer to the brain, assuming that the higher expression of some of these genes favors survival or growth. Similarly, 205 genes reached a maximum expression ≥2-fold higher in the second passage on brain tissue, and there are small molecule inhibitors for 54 of these, suggesting a potential strategy to treat slightly more advanced brain-metastatic cancer, still prior to the rapid growth phase. Fifty-two (52) genes reached their maximum expression of ≥2-fold in the third passage, and there are inhibitors for 13 of these. Similarly, 201 genes were up-regulated by ≥2-fold in at least one time point when the breast cancer cells were co-cultivated with bone marrow, 61 of which have known inhibitors. 204 genes were up-regulated by ≥2-fold in at least one time point when the breast cancer cells were co-cultivated with lung, for which there are also, coincidentally, 61 inhibitors. There were 101 genes up-regulated by ≥2-fold in both the bone marrow-adapted and lung-adapted cells, and there are inhibitors for 40 of these. Many of these inhibitors correspond to drugs already approved to treat cancer, while many others are currently used to treat maladies other than cancer, and might be repurposed to prevent or treat breast cancer metastasis.

The tissue-dependence of gene expression observed in cells co-cultured briefly with various tissues, especially when the changes revert to parental levels in later passages, indicates a potential shortcoming in drug screening strategies for cytostatic and cytotoxic agents that use tumor-derived cell lines growing *in vitro* that have been maintained in culture for many generations. It would be surprising to discover that the changes induced *in vivo* were stable for many generations *in vitro*. In cases in which such induced genes imparted a growth advantage to the cells growing *in vivo*, one would generally not expect these advantages to persist during the *in vitro* stage of our protocol. Therefore, drugs that inhibit such genes may influence growth in the tissue environment, but would not be expected to influence growth *in vitro*, and would be missed in screens that do not have an *in vivo* component.

The large number of tumor cells grafted onto tissues in the dorsal skinfold chambers in these experiments suggests a potentially significant caveat: the microenvironment experienced by the grafted cells is partially determined by the tumor cells, themselves, whereas a genuine extravasated tumor cell exists as a single cell possibly through a very long period of dormancy. Therefore, by using a large number of cells, we increase the chances of one or more clones surviving and growing, but sacrifice fidelity with the true biological situation. Also, these studies are based on a small number of adaptation experiments, and therefore cannot be generalized without caution. However, the expression of stress- and tissue-specific genes in early stages of adaptation, followed by apparent adaptation to the novel environment, followed by more demanding cell-physiological processes such as morphological reticulation and growth, conform to the prevailing general notions of how cancer, particularly metastatic cancer, must progress. Also, while cells of the experimental tumors are serially transplanted in this experimental design, the organ tissue is not, thereby precluding any long-term adaptive response on the part of the microenvironment in these experiments.

A concerted effort to identify the time-resolved changes that occur during cancer progression in animal models may provide new insights into treatment strategies especially with regard to gene expression changes that occur early during metastasis and therefore represent likely drug targets for metastasis prevention or delay. Our experiments do not exclude the likely possibility that some of the changes in gene expression we observe may occur prior to *bona fide* metastasis when the tumor cells are in circulation.

## Materials and Methods

### Ethics statement

All procedures involving mice were approved by the Institutional Animal Care and Use Committee of Explora Biolabs (San Diego, CA), and performed under Protocol EB09-022. Experiments were carried out in accordance with recommendations in the Guide for the Care and Use of Laboratory Animals of the National Institutes of Health. Every effort was made to minimize animal suffering, anxiety, and discomfort.

### Intravital video microscopy

Platinum chambers were fitted in the dorsal skinfold of female nude mice by surgery as described extensively in [13], [14]. Brain, bone marrow and lung tissue were obtained from donor mice (C57BL/6), minced with a razor blade and ∼10 micrograms of minced tissue were introduced into each dorsal skinfold chamber. These tissues were allowed to revascularize for 7–10 days, to form “pseudo-brain”, “pseudo-bone marrow”, and “pseudo-lung”. Spheroids were prepared as described previously [13], [14], from the HER2-positive mouse breast cancer cell line clone N202.1A, which were derived from mammary carcinomas of FVB-NeuN #202 mice (H-2q), expressing a rat HER-2/neu proto-oncogene transgene, controlled by the MMTV promoter [55]. The N202 cells were further modified to express enhanced green fluorescent protein (eGFP) from a stably integrated H2B-GFP construct [15]. These N202 spheroids were grafted on top of the revascularized bone marrow, lung tissue, or brain tissue. Growth of the N202 cells expressing H2B-GFP was followed via the GFP using fluorescence microscopy through the window of the dorsal skin fold chamber. After 21 days, the developing tumors were removed from the chambers and placed in cell culture for 2-4 weeks, depending on the rate of cell growth. Cells expressing GFP were enriched by selection with G418, and again expanded in culture for 2-4 weeks. New spheroids were again prepared and a new cycle of grafting into freshly prepared dorsal skin fold chambers was initiated. This was repeated for a total of 4 cycles. To determine cell growth rates, each passage of N202 for the three tissues were seeded *in vitro* at ∼30000 cells in triplicate and counted every three days during two weeks using a hemocytometer. The mean was calculated for each time point, using 6 or more values.

### Gene expression analysis

At each cycle, cells were then sorted based on GFP, RNA was harvested from these highly purified cultures, and gene expression analysis was performed on Affymetrix GeneChip mouse gene 1.0 St array, following the manufacturer's protocol for target preparation, hybridization, washing and scanning. The cells were grown to 60–70% confluence, and total RNA was extracted using an RNeasy Mini Kit (Qiagen, Valencia, CA) following the manufacturer's instructions. RNA concentration was determined by spectroscopy, and integrity was assessed qualitatively by agarose gel electrophoresis. 100 ng of total RNA were used for target preparation.

The parental cell line served as a common control. Technical replicates starting with cells in culture for the common control (P0) and for the cells harvested at the four subsequent passages (P1-4) for cells adapted to brain tissue were performed. Cells harvested from bone marrow and lung tissue were analyzed once per time point. Gene expression data were processed using the RMA (Robust Multiarray Average) [56] algorithm in the Affymetrix Expression Console Software package (**Fig. S7**). Data were analyzed using routines written in R [57]. Genes were filtered for those having signal intensities exceeding 95% of the signals from negative hybridization controls, and this yielded 10,522 positive probe sets (**Fig. S8**). Gene expression data are deposited with the NCBI Gene Expression Omnibus (GSE54626).

### Data analysis procedures

Ratios were obtained by dividing the signal log ratios of intensities obtained for each gene after each of the four serial passages described above, creating a 5 element vector for each gene of the form (0, x_1_, x_2_, x_3_, x_4_) where x_n_ are the ratios for each passage, and these vectors were sorted into k-means groups. When using k-means, a decision must be made regarding the number of groups, N, and there is no rigorously correct way to do this. One approach is to set a maximum acceptable fitting error (i.e. the ‘sum of squares within’), another is to track the error assuming different numbers of groups and to choose a number of groups beyond which the error no longer decreases rapidly (i.e. identify an “elbow”), and a third is to try different numbers of groups and test them post hoc for statistically significant biological correlations; we used the latter. We found statistically significant correlations with GO biological process terms using different assumptions for N. P-values were determined by Fisher's exact test and corrected to yield the false discovery rate (FDR) according to Benjamini and Hochberg [19]. This was done assuming N = 9, 16, 25, 36, 49, and 64, where N corresponds to the number of k-means groups. Duplicate microarrays were performed for the parental (unadapted) cell line (**Fig. S9** ****) and each time point of the brain experiment (**Fig. S10** ), and duplicate signal measurements were averaged after RMA. For the bone marrow and lung experiments, a single microarray experiment was done for each time point.

Each gene was matched to GO Biological Process Terms. Annotations were the union of the original Affymetrix GeneChip mouse gene1.0 St array provided by the Affymetrix Expression Console software and annotations from Mouse Genome Informatics (http://www.informatics.jax.org). For each value of N, the frequency of each GO Biological Process Term within N k-means groups was calculated. Values for the expected frequencies were calculated based on the number of genes in each group. For each GO term and for each group, a 2×2 contingency table was generated containing the frequency of genes annotated and not annotated with the term within the k-means group, compared with the total number of genes annotated and not annotated with the term. Fisher's exact test was performed on each contingency table to give a list of terms and p-values associated with k-means group. This is a multiple testing situation, so false discovery rates (qval) were calculated by sorting p-values from largest to smallest and dividing by their rank, according to Benjamini and Hochberg [19]. These lists were then sorted by qval from smallest to largest and stored in a MySQL database. A web interface was built to access this database. Genes and GO terms are cross-referenced by links to NCBI/Gene and AmiGO (http://amigo.geneontology.org), respectively. We used Therapeutic Targets Database drug-gene database (http://bidd.nus.edu.sg/group/ttd/), The Drug Gene Interaction Database (http://dgidb.genome.wustl.edu/), and biodbnet (http://biodbnet.abcc.ncifcrf.gov/); the latter was used to recover official gene symbols for cross-species comparison. We include other drugs and inhibitors gleaned from the literature. These are usually referenced via a Pubmed link. Note that identical or similar drug effects across species cannot be assured.

## Supporting Information

Figure S1 Population doubling per day in vitro and in vivo. (A, B, C) In vitro rates of population doubling per day (PD/D) for cells grown from in vivo passages 0–4 for brain, bone marrow, and lung tissue, respectively, and 3 independent measurements per time point. Error bars represent one standard deviation. (D, E, F) In vivo growth rates, 2 animals per measurement. (G, H, I) Correlation between in vitro and in vitro growth rates; r is Pearson's correlation. (Note that the cells of the final time point were not transferred into the chamber, and therefore the second row has one fewer time point than the first).
(TIF)

Figure S2 Adaptation of N202 cells to brain, bone marrow and lung tissue. N = 25. Gray lines represent 4-fold changes. Horizontally, positions represent sequential in vivo passages, P0, P1, P2, P3, P4, and gene expression analysis was performed on cells expanded in vitro from each of these passages. K-means clustering for (**A**) brain tissue, (**B**) bone marrow tissue, (**C**) lung tissue. (A) corresponds to Figure 2 in the text and is included here for convenience. As in Figure 2, the central black line in each frame represents no change, i.e. 1-fold changes, and the gray lines represent 4-fold increases or decreases, relative to the parental cell line. Horizontally, the five positions along the x-axis represent sequential in vivo passages, P0 (i.e. parental cells), P1, P2, P3, P4, and gene expression analysis was performed on cells expanded in vitro from each of these passages. (B) and (C) represent the corresponding clusters for bone marrow and lung.
(TIF)

Figure S3 Cell morphology during adaptation to brain tissue. Cell morphology during adaptation to brain tissue varies considerably. Three example fields of cells at each adaptive stage are shown. (**P0**) The parental cell line has short dendritic projections. (**P1**) Cells after the first passage become slightly less complex in shape. (**P2**) After the second passage, cells have very few dendritic projections. (**P3**) Cells of P3 have numerous, stumpy dendritic extensions, and this phenotype is more pronounced in (**P4**).
(TIF)

Figure S4 (A) Robust neovascularization in P1. Bright field images showing that neovascularization of the developing tumor (evident as a raised, bright nodule) becomes robust in the first passage (P1) in all three tissues, brain (bn), bone marrow (bm), and lung (ln). Pressure against the glass due to rapid growth reduces circulation in P1 ln in the picture on the right. (**B**) **Robust neovascularization in all passages**. Bright field images of N202 cells growing in three successive passages (P1–3) of independently adapting lineages on brain tissue, illustrating that vascularization is robust for all passages.
(TIF)

Figure S5 Concordance between clinical brain metastasis and this model system. These histograms show the correspondence between human metastatic breast cancer to the brain and the transition from P0 to P1, 2, 3 and 4 when N202 cells are trained to grow on brain tissue in the dorsal skinfold chamber. This analysis is based on gene expression data from Palmieri et al. [45], in which genes that are up-regulated or down-regulated in breast cancer metastasis to the brain are described. 21 out of 56 genes that were significantly different in metastasis vs. primary tumors identified in [45] were represented by orthologs in our data. We devised a statistic for concordance, c/n, in which c is the number of genes that change in the same direction in the two experiments and n is the number of genes being compared, i.e. n = 21. 18 out of 21 (i.e. c/n = 18/21 = 86%) of these genes that were significantly different in primary tumors vs. metastatic tumors were altered in the same direction in our data in the transition P0→P2, with p = 0.0002. A ratio near 0.86 could occur by chance if ∼86% of the changes in gene expression observed in our data corresponded to down-regulation; therefore, p-values were determined by comparing the observed c/n (e.g. 18/21≈0.86 for P0→P2) with c/n for 10,000 randomly selected sets of 21 genes. These results are summarized as histograms. The p-value of p = 0.0002 (or with a Bonferonni correction; p = 0.0008) for the P0→P2 transition indicates strong concordance of clinical brain-metastatic breast cancer and the P0→P2 transition in our experimental model. This similarity was not seen in the transitions P0→P1, P0→P3, or P0→P4, although the transition P0→P1 nearly reaches a significant p-value. The red vertical lines represent the observed value for c/n and the p-values are indicated.
(TIF)

Figure S6 Biological replicate of transplantation of N202 tumor cell spheroids to brain tissue. This biological replicate was performed exactly as the initial experiment. There is no reason to anticipate that the rate of evolution in two independent experiments would be identical. Therefore, we sought to match the two sequential passages repP1 and repP2 from the replicate experiment to the appropriate position in the original time course, P0-P4. To achieve this, a spline curve was fit to every gene in the plot of passage vs. RMA-normalized gene expression signal. We used these spline curves to fill a matrix of interpolated intermediate values for each gene at intermediate passages, e.g. P0, P0.002, P0.004,…, P4.000. We then calculated the sum of absolute differences between repP1 and every column in this matrix, and assigned repP1 to the column in the matrix where this statistic was minimum. The position assignment was based on 1000 random bootstrap samples of 100 randomly sampled genes. This was repeated for repP2. Histograms indicated that repP1 and repP2 fit best at P2.65 (blue) and P3.82 (red), respectively. The grey bar corresponds to original P2 as an algorithm check.
(TIF)

Figure S7 Box plot showing the results of robust multiarray averaging (RMA). The robust multiarray average implemented in Affymetrix Expression Array Console Software. UT = Untrained, BN = brain, BNr = brain technical repeat, BM = bone marrow, LN = lung, BR = breast. Boxes are bounded at the first and third quartiles. Wiskers are at 1.5 times the range.
(TIF)

Figure S8 Histogram of PM signals on Affymetrix arrays. Yellow = all PM (perfect match) scores, Red = negative hybridization controls, Blue = positive hybridization “spike-in” controls provided by the manufacturer. The vertical red line is placed at the position below which are 95% of the negative controls. Genes were included for which the signal exceeded this threshold for at least one trained or untrained cell line.
(TIF)

Figure S9 Scatterplot of RMA-normalized untrained control replicates. UT1 and UT2 microarrays were repeated beginning with two independent cell cultures and corresponding RNA preparations, and plotted as a scatterplot. This plot provides a visual demonstration of reproducibility.
(TIF)

Figure S10 Technical replicate reproducibility. Technical replicates of microarrays for cells trained to grow on brain tissue, beginning with independent cell cultures and corresponding RNA preparations.
(TIF)

Table S1 Significant GO Biological Process Terms related to “transport” associated with genes that are UP-REGULATED upon initial contact with Brain, Bone Marrow, and Lung Tissues.
(DOCX)

Table S2 Genes associated with GO Biological Process terms discussed in the text.
(DOCX)

Table S3 Genes strongly induced or repressed in different passages in brain tissue.
(DOCX)

## References

[1] Kobayashi K, Sawada K, Yamamoto H, Wada S, Saiga H, et al (2003) Maternal macho-1 is an intrinsic factor that makes cell response to the same FGF signal differ between mesenchyme and notochord induction in ascidian embryos. Development 130: 5179–5190. 12954719 10.1242/dev.00732

[2] Takahashi M, Palmer TD, Takahashi J, Gage FH (1998) Widespread integration and survival of adult-derived neural progenitor cells in the developing optic retina. Mol Cell Neurosci 12: 340–348. 9888988 10.1006/mcne.1998.0721

[3] Bonfanti P, Claudinot S, Amici AW, Farley A, Blackburn CC, et al (2010) Microenvironmental reprogramming of thymic epithelial cells to skin multipotent stem cells. Nature 466: 978–982. 20725041 10.1038/nature09269

[4] Tlsty TD, Coussens LM (2006) Tumor stroma and regulation of cancer development. Annu Rev Pathol 1: 119–150. 18039110 10.1146/annurev.pathol.1.110304.100224

[5] Joyce JA, Pollard JW (2009) Microenvironmental regulation of metastasis. Nat Rev Cancer 9: 239–252. 19279573 10.1038/nrc2618 PMC3251309

[6] Gupta GP, Massague J (2006) Cancer metastasis: building a framework. Cell 127: 679–695. 17110329 10.1016/j.cell.2006.11.001

[7] Hanahan D, Weinberg RA (2011) Hallmarks of cancer: the next generation. Cell 144: 646–674. 21376230 10.1016/j.cell.2011.02.013

[8] Yoneda T, Williams PJ, Hiraga T, Niewolna M, Nishimura R (2001) A bone-seeking clone exhibits different biological properties from the MDA-MB-231 parental human breast cancer cells and a brain-seeking clone in vivo and in vitro. J Bone Miner Res 16: 1486–1495. 11499871 10.1359/jbmr.2001.16.8.1486

[9] Bos PD, Zhang XH, Nadal C, Shu W, Gomis RR, et al (2009) Genes that mediate breast cancer metastasis to the brain. Nature 459: 1005–1009. 19421193 10.1038/nature08021 PMC2698953

[10] Aslakson CJ, Miller FR (1992) Selective events in the metastatic process defined by analysis of the sequential dissemination of subpopulations of a mouse mammary tumor. Cancer Res 52: 1399–1405. 1540948

[11] Yang J, Mani SA, Donaher JL, Ramaswamy S, Itzykson RA, et al (2004) Twist, a master regulator of morphogenesis, plays an essential role in tumor metastasis. Cell 117: 927–939. 15210113 10.1016/j.cell.2004.06.006

[12] Giancotti FG (2013) Mechanisms governing metastatic dormancy and reactivation. Cell 155: 750–764. 24209616 10.1016/j.cell.2013.10.029 PMC4354734

[13] Oh P, Borgstrom P, Witkiewicz H, Li Y, Borgstrom BJ, et al (2007) Live dynamic imaging of caveolae pumping targeted antibody rapidly and specifically across endothelium in the lung. Nat Biotechnol 25: 327–337. 17334358 10.1038/nbt1292 PMC1979160

[14] Frost GI, Lustgarten J, Dudouet B, Nyberg L, Hartley-Asp B, et al (2005) Novel syngeneic pseudo-orthotopic prostate cancer model: vascular, mitotic and apoptotic responses to castration. Microvasc Res 69: 1–9. 15797254 10.1016/j.mvr.2004.10.001

[15] Cuadros C, Dominguez AL, Frost GI, Borgstrom P, Lustgarten J (2003) Cooperative effect between immunotherapy and antiangiogenic therapy leads to effective tumor rejection in tolerant Her-2/neu mice. Cancer Res 63: 5895–5901. 14522915

[16] Dulbecco R, Armstrong B (1988) Stochastic development of invasive potential in rat mammary tumors induced by N-methyl-N-nitrosourea. Proc Natl Acad Sci U S A 85: 8659–8663. 2460874 10.1073/pnas.85.22.8659 PMC282519

[17] Chan MM, Lu X, Merchant FM, Iglehart JD, Miron PL (2007) Serial transplantation of NMU-induced rat mammary tumors: a model of human breast cancer progression. Int J Cancer 121: 474–485. 17405122 10.1002/ijc.22684

[18] Perez-Llamas C, Lopez-Bigas N (2011) Gitools: analysis and visualisation of genomic data using interactive heat-maps. PLoS One 6: e19541. 21602921 10.1371/journal.pone.0019541 PMC3094337

[19] Benjamini Y, Hochberg Y (1995) Controlling the false discovery rate: a practical and powerful approach to multiple testing. Journal of the Royal Statistical Society Series B 57: 289–300.

[20] Abbott NJ, Ronnback L, Hansson E (2006) Astrocyte-endothelial interactions at the blood-brain barrier. Nat Rev Neurosci 7: 41–53. 16371949 10.1038/nrn1824

[21] Steeg PS, Bevilacqua G, Kopper L, Thorgeirsson UP, Talmadge JE, et al (1988) Evidence for a novel gene associated with low tumor metastatic potential. J Natl Cancer Inst 80: 200–204. 3346912 10.1093/jnci/80.3.200

[22] Parsons JT, Horwitz AR, Schwartz MA (2010) Cell adhesion: integrating cytoskeletal dynamics and cellular tension. Nat Rev Mol Cell Biol 11: 633–643. 20729930 10.1038/nrm2957 PMC2992881

[23] Kaufman RJ (1999) Stress signaling from the lumen of the endoplasmic reticulum: coordination of gene transcriptional and translational controls. Genes Dev 13: 1211–1233. 10346810 10.1101/gad.13.10.1211

[24] Benosman S, Ravanan P, Correa RG, Hou YC, Yu M, et al (2013) Interleukin-1 receptor-associated kinase-2 (IRAK2) is a critical mediator of endoplasmic reticulum (ER) stress signaling. PLoS One 8: e64256. 23724040 10.1371/journal.pone.0064256 PMC3665826

[25] McCullough KD, Martindale JL, Klotz LO, Aw TY, Holbrook NJ (2001) Gadd153 sensitizes cells to endoplasmic reticulum stress by down-regulating Bcl2 and perturbing the cellular redox state. Mol Cell Biol 21: 1249–1259. 11158311 10.1128/MCB.21.4.1249-1259.2001 PMC99578

[26] Cory S, Adams JM (2002) The Bcl2 family: regulators of the cellular life-or-death switch. Nat Rev Cancer 2: 647–656. 12209154 10.1038/nrc883

[27] Zhang P, Hu X, Xu X, Chen Y, Bache RJ (2011) Dimethylarginine dimethylaminohydrolase 1 modulates endothelial cell growth through nitric oxide and Akt. Arterioscler Thromb Vasc Biol 31: 890–897. 21212404 10.1161/ATVBAHA.110.215640 PMC3064458

[28] Eilken HM, Adams RH (2010) Dynamics of endothelial cell behavior in sprouting angiogenesis. Curr Opin Cell Biol 22: 617–625. 20817428 10.1016/j.ceb.2010.08.010

[29] Overgaard K, Nielsen OB, Flatman JA, Clausen T (1999) Relations between excitability and contractility in rat soleus muscle: role of the Na+-K+ pump and Na+/K+ gradients. J Physiol 518 (Pt 1): 215–225. 10.1111/j.1469-7793.1999.0215r.x PMC2269417 10373703

[30] Brochner CB, Johansen JS, Larsen LA, Bak M, Mikkelsen HB, et al (2012) YKL-40 is differentially expressed in human embryonic stem cells and in cell progeny of the three germ layers. J Histochem Cytochem 60: 188–204. 22140133 10.1369/0022155411433331 PMC3351129

[31] Kruse AC, Hu J, Pan AC, Arlow DH, Rosenbaum DM, et al (2012) Structure and dynamics of the M3 muscarinic acetylcholine receptor. Nature 482: 552–556. 22358844 10.1038/nature10867 PMC3529910

[32] Molinari F, Rio M, Meskenaite V, Encha-Razavi F, Auge J, et al (2002) Truncating neurotrypsin mutation in autosomal recessive nonsyndromic mental retardation. Science 298: 1779–1781. 12459588 10.1126/science.1076521

[33] Hagglund MG, Sreedharan S, Nilsson VC, Shaik JH, Almkvist IM, et al (2011) Identification of SLC38A7 (SNAT7) protein as a glutamine transporter expressed in neurons. J Biol Chem 286: 20500–20511. 21511949 10.1074/jbc.M110.162404 PMC3121473

[34] Gros-Louis F, Dupre N, Dion P, Fox MA, Laurent S, et al (2007) Mutations in SYNE1 lead to a newly discovered form of autosomal recessive cerebellar ataxia. Nat Genet 39: 80–85. 17159980 10.1038/ng1927

[35] Saadat L, Dupree JL, Kilkus J, Han X, Traka M, et al (2010) Absence of oligodendroglial glucosylceramide synthesis does not result in CNS myelin abnormalities or alter the dysmyelinating phenotype of CGT-deficient mice. Glia 58: 391–398. 19705459 10.1002/glia.20930 PMC2807477

[36] Lombard Z, Park C, Makova KD, Ramsay M (2011) A computational approach to candidate gene prioritization for X-linked mental retardation using annotation-based binary filtering and motif-based linear discriminatory analysis. Biol Direct 6: 30. 21668950 10.1186/1745-6150-6-30 PMC3142252

[37] Metz M, Gassmann M, Fakler B, Schaeren-Wiemers N, Bettler B (2011) Distribution of the auxiliary GABAB receptor subunits KCTD8, 12, 12b, and 16 in the mouse brain. J Comp Neurol 519: 1435–1454. 21452234 10.1002/cne.22610

[38] Funayama M, Hasegawa K, Kowa H, Saito M, Tsuji S, et al (2002) A new locus for Parkinson's disease (PARK8) maps to chromosome 12p11.2-q13.1. Ann Neurol 51: 296–301. 11891824 10.1002/ana.10113

[39] Cases O, Seif I, Grimsby J, Gaspar P, Chen K, et al (1995) Aggressive behavior and altered amounts of brain serotonin and norepinephrine in mice lacking MAOA. Science 268: 1763–1766. 7792602 10.1126/science.7792602 PMC2844866

[40] Straub C, Zhang W, Howe JR (2011) Neto2 modulation of kainate receptors with different subunit compositions. J Neurosci 31: 8078–8082. 21632929 10.1523/JNEUROSCI.0024-11.2011 PMC3118549

[41] Rojas P, Joodmardi E, Hong Y, Perlmann T, Ogren SO (2007) Adult mice with reduced Nurr1 expression: an animal model for schizophrenia. Mol Psychiatry 12: 756–766. 17457314 10.1038/sj.mp.4001993

[42] Kim SY, Yasuda S, Tanaka H, Yamagata K, Kim H (2011) Non-clustered protocadherin. Cell Adh Migr 5: 97–105. 21173574 10.4161/cam.5.2.14374 PMC3084973

[43] Horiuchi Y, Iida S, Koga M, Ishiguro H, Iijima Y, et al (2012) Association of SNPs linked to increased expression of SLC1A1 with schizophrenia. Am J Med Genet B Neuropsychiatr Genet 159B: 30–37. 22095641 10.1002/ajmg.b.31249

[44] Han HX, Geng JG (2011) Over-expression of Slit2 induces vessel formation and changes blood vessel permeability in mouse brain. Acta Pharmacol Sin 32: 1327–1336. 21986575 10.1038/aps.2011.106 PMC3891311

[45] Palmieri D, Fitzgerald D, Shreeve SM, Hua E, Bronder JL, et al (2009) Analyses of resected human brain metastases of breast cancer reveal the association between up-regulation of hexokinase 2 and poor prognosis. Mol Cancer Res 7: 1438–1445. 19723875 10.1158/1541-7786.MCR-09-0234 PMC2746883

[46] Moreira LO, El Kasmi KC, Smith AM, Finkelstein D, Fillon S, et al (2008) The TLR2-MyD88-NOD2-RIPK2 signalling axis regulates a balanced pro-inflammatory and IL-10-mediated anti-inflammatory cytokine response to Gram-positive cell walls. Cell Microbiol 10: 2067–2077. 18549453 10.1111/j.1462-5822.2008.01189.x PMC4966886

[47] Miles RR, Sluka JP, Halladay DL, Santerre RF, Hale LV, et al (2000) ADAMTS-1: A cellular disintegrin and metalloprotease with thrombospondin motifs is a target for parathyroid hormone in bone. Endocrinology 141: 4533–4542. 11108265 10.1210/endo.141.12.7817

[48] Lu X, Kang Y (2009) Metalloproteinases and osteoblast EGFR signaling in osteolytic bone metastasis of breast cancer. Cell Cycle 8: 3804–3805. 19934661 10.4161/cc.8.23.10104

[49] Sabrautzki S, Rubio-Aliaga I, Hans W, Fuchs H, Rathkolb B, et al (2012) New mouse models for metabolic bone diseases generated by genome-wide ENU mutagenesis. Mamm Genome 23: 416–430. 22527485 10.1007/s00335-012-9397-z PMC3401305

[50] Khoo ML, Tao H, Meedeniya AC, Mackay-Sim A, Ma DD (2011) Transplantation of neuronal-primed human bone marrow mesenchymal stem cells in hemiparkinsonian rodents. PLoS One 6: e19025. 21625433 10.1371/journal.pone.0019025 PMC3100305

[51] Velasco J, Zarrabeitia MT, Prieto JR, Perez-Castrillon JL, Perez-Aguilar MD, et al (2011) Wnt pathway genes in osteoporosis and osteoarthritis: differential expression and genetic association study. Osteoporos Int 21: 109–118. 10.1007/s00198-009-0931-0 19373426

[52] Johnston J, Ramos-Valdes Y, Stanton LA, Ladhani S, Beier F, et al (2010) Human stanniocalcin-1 or -2 expressed in mice reduces bone size and severely inhibits cranial intramembranous bone growth. Transgenic Res 19: 1017–1039. 20174869 10.1007/s11248-010-9376-7

[53] Palmieri D, Lockman PR, Thomas FC, Hua E, Herring J, et al (2009) Vorinostat inhibits brain metastatic colonization in a model of triple-negative breast cancer and induces DNA double-strand breaks. Clin Cancer Res 15: 6148–6157. 19789319 10.1158/1078-0432.CCR-09-1039 PMC7356672

[54] Scheel C, Onder T, Karnoub A, Weinberg RA (2007) Adaptation versus selection: the origins of metastatic behavior. Cancer Res 67: 11476–11479 discussion 11479–11480. 18089773 10.1158/0008-5472.CAN-07-1653

[55] Astolfi A, Landuzzi L, Nicoletti G, De Giovanni C, Croci S, et al (2005) Gene expression analysis of immune-mediated arrest of tumorigenesis in a transgenic mouse model of HER-2/neu-positive basal-like mammary carcinoma. Am J Pathol 166: 1205–1216. 15793299 10.1016/S0002-9440(10)62339-5 PMC1602398

[56] Irizarry RA, Hobbs B, Collin F, Beazer-Barclay YD, Antonellis KJ, et al (2003) Exploration, normalization, and summaries of high density oligonucleotide array probe level data. Biostatistics 4: 249–264. 12925520 10.1093/biostatistics/4.2.249

[57] R Core Team (2012) R: A language and environment for statistical computing. R Foundation for Statistical Computing, Vienna, Austria, http://wwwR-projectorg

