# Supplementary figures and images for: Effects of Different Tissue Microenvironments on Gene Expression in Breast Cancer Cells
Source: PLoS One. 2014 Jul 8;9(7):e101160. doi: 10.1371/journal.pone.0101160 (PMC4086928; doi:10.1371/journal.pone.0101160)

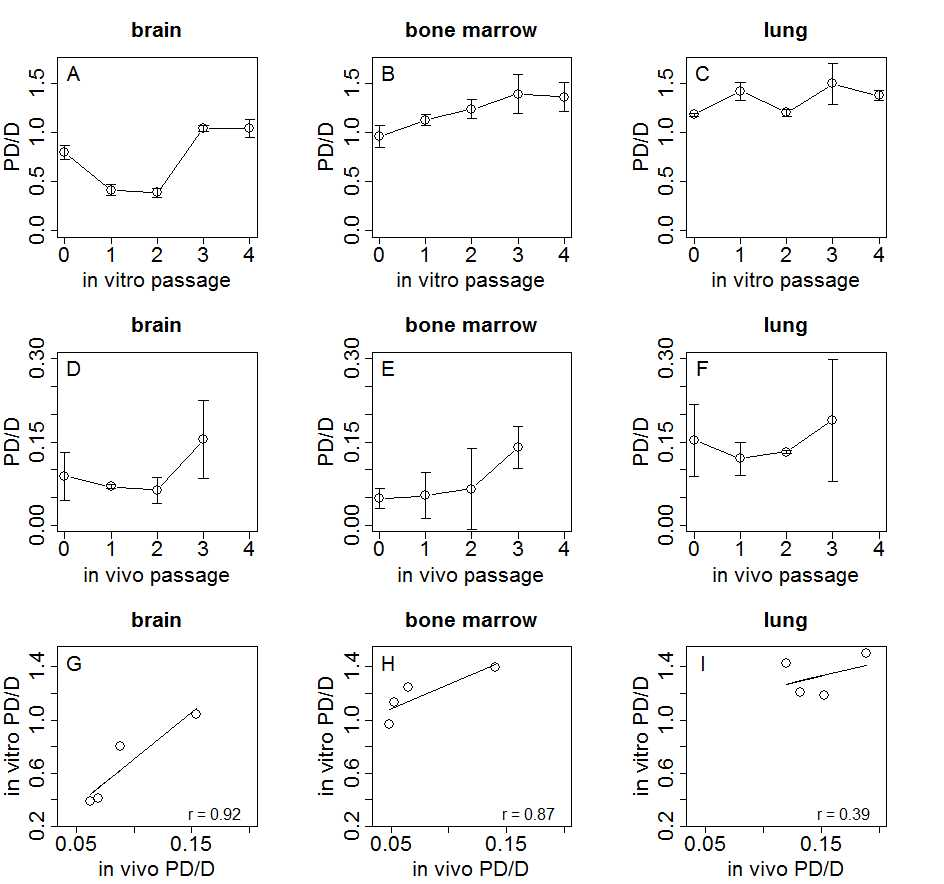

Supplement: Figure S1 — Population doubling per day in vitro and in vivo. (A, B, C) In vitro rates of population doubling per day (PD/D) for cells grown from in vivo passages 0–4 for brain, bone marrow, and lung tissue, respectively, and 3 independent measurements per time point. Error bars represent one standard deviation. (D, E, F) In vivo growth rates, 2 animals per measurement. (G, H, I) Correlation between in vitro and in vitro growth rates; r is Pearson's correlation. (Note that the cells of the final time point were not transferred into the chamber, and therefore the second row has one fewer time point than the first). (TIF) [file pone.0101160.s001.tif]

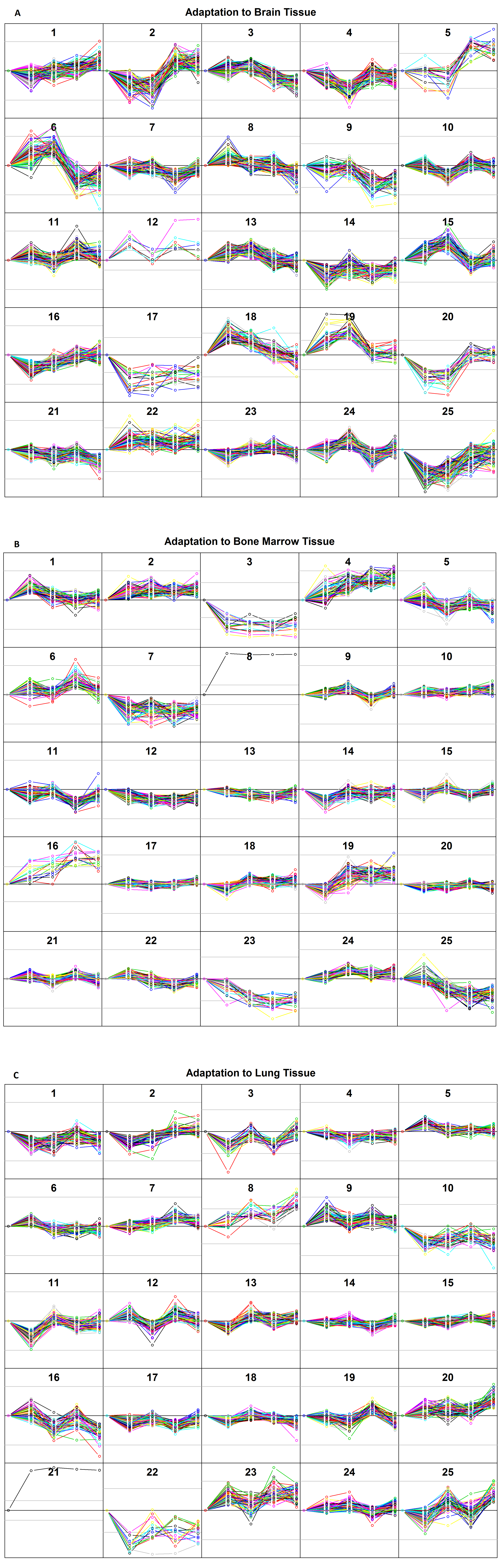

Supplement: Figure S2 — Adaptation of N202 cells to brain, bone marrow and lung tissue. N = 25. Gray lines represent 4-fold changes. Horizontally, positions represent sequential in vivo passages, P0, P1, P2, P3, P4, and gene expression analysis was performed on cells expanded in vitro from each of these passages. K-means clustering for (A) brain tissue, (B) bone marrow tissue, (C) lung tissue. (A) corresponds to Figure 2 in the text and is included here for convenience. As in Figure 2, the central black line in each frame represents no change, i.e. 1-fold changes, and the gray lines represent 4-fold increases or decreases, relative to the parental cell line. Horizontally, the five positions along the x-axis represent sequential in vivo passages, P0 (i.e. parental cells), P1, P2, P3, P4, and gene expression analysis was performed on cells expanded in vitro from each of these passages. (B) and (C) represent the corresponding clusters for bone marrow and lung. (TIF) [file pone.0101160.s002.tif]

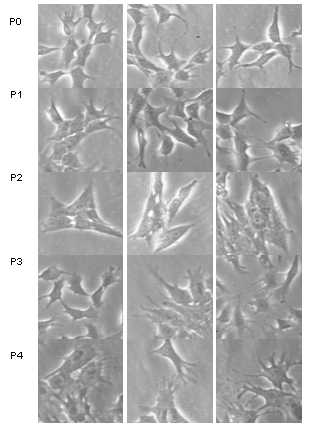

Supplement: Figure S3 — Cell morphology during adaptation to brain tissue. Cell morphology during adaptation to brain tissue varies considerably. Three example fields of cells at each adaptive stage are shown. (P0) The parental cell line has short dendritic projections. (P1) Cells after the first passage become slightly less complex in shape. (P2) After the second passage, cells have very few dendritic projections. (P3) Cells of P3 have numerous, stumpy dendritic extensions, and this phenotype is more pronounced in (P4). (TIF) [file pone.0101160.s003.tif]

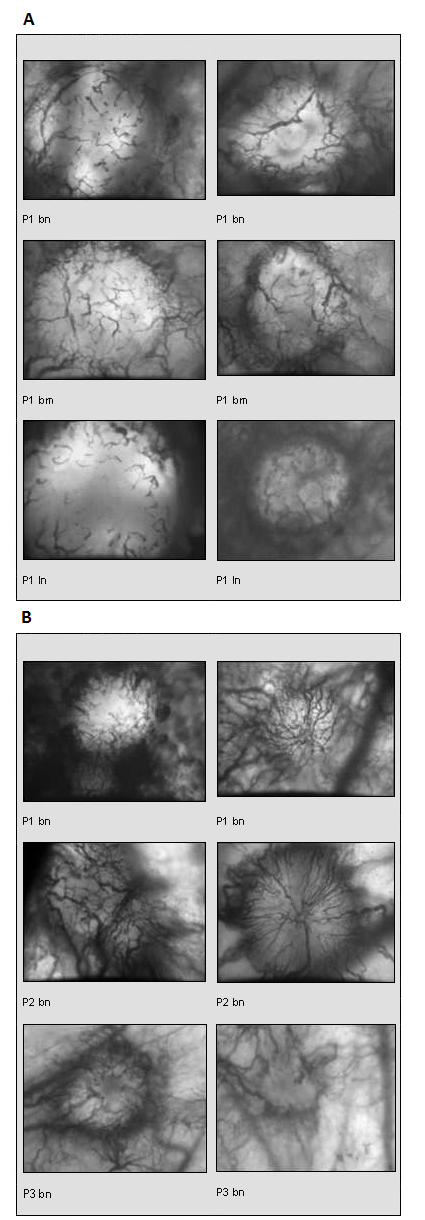

Supplement: Figure S4 — (A) Robust neovascularization in P1. Bright field images showing that neovascularization of the developing tumor (evident as a raised, bright nodule) becomes robust in the first passage (P1) in all three tissues, brain (bn), bone marrow (bm), and lung (ln). Pressure against the glass due to rapid growth reduces circulation in P1 ln in the picture on the right. (B) Robust neovascularization in all passages. Bright field images of N202 cells growing in three successive passages (P1–3) of independently adapting lineages on brain tissue, illustrating that vascularization is robust for all passages. (TIF) [file pone.0101160.s004.tif]

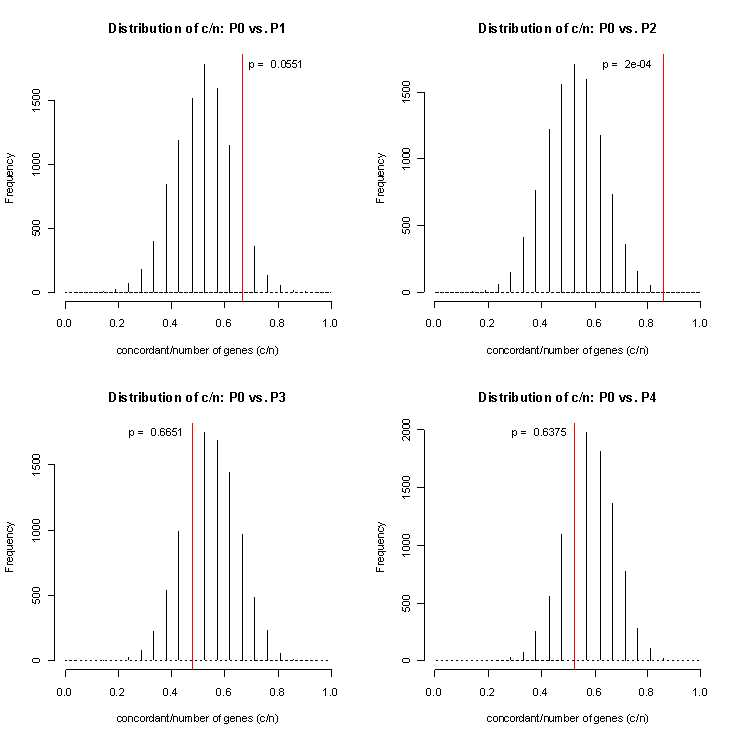

Supplement: Figure S5 — Concordance between clinical brain metastasis and this model system. These histograms show the correspondence between human metastatic breast cancer to the brain and the transition from P0 to P1, 2, 3 and 4 when N202 cells are trained to grow on brain tissue in the dorsal skinfold chamber. This analysis is based on gene expression data from Palmieri et al. [45], in which genes that are up-regulated or down-regulated in breast cancer metastasis to the brain are described. 21 out of 56 genes that were significantly different in metastasis vs. primary tumors identified in [45] were represented by orthologs in our data. We devised a statistic for concordance, c/n, in which c is the number of genes that change in the same direction in the two experiments and n is the number of genes being compared, i.e. n = 21. 18 out of 21 (i.e. c/n = 18/21 = 86%) of these genes that were significantly different in primary tumors vs. metastatic tumors were altered in the same direction in our data in the transition P0→P2, with p = 0.0002. A ratio near 0.86 could occur by chance if ∼86% of the changes in gene expression observed in our data corresponded to down-regulation; therefore, p-values were determined by comparing the observed c/n (e.g. 18/21≈0.86 for P0→P2) with c/n for 10,000 randomly selected sets of 21 genes. These results are summarized as histograms. The p-value of p = 0.0002 (or with a Bonferonni correction; p = 0.0008) for the P0→P2 transition indicates strong concordance of clinical brain-metastatic breast cancer and the P0→P2 transition in our experimental model. This similarity was not seen in the transitions P0→P1, P0→P3, or P0→P4, although the transition P0→P1 nearly reaches a significant p-value. The red vertical lines represent the observed value for c/n and the p-values are indicated. (TIF) [file pone.0101160.s005.tif]

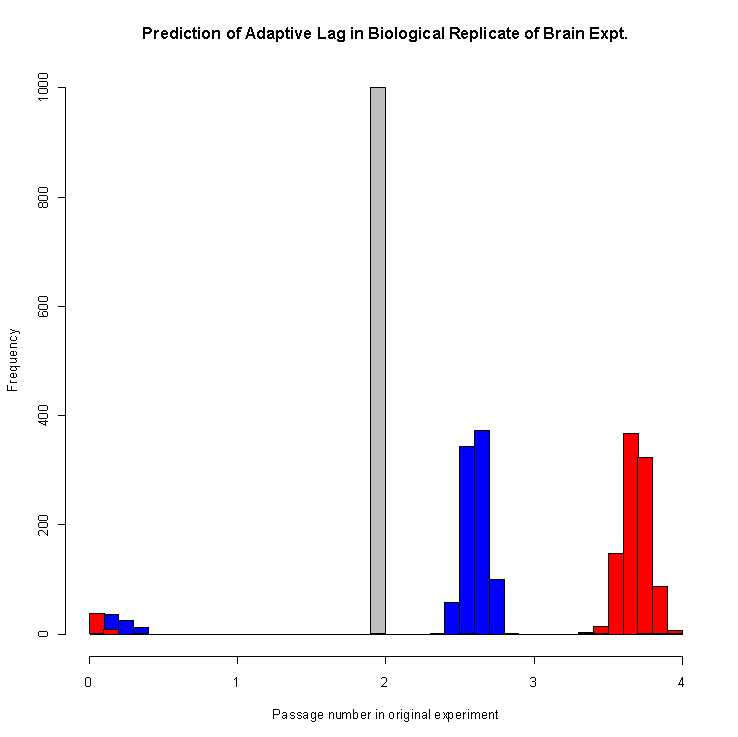

Supplement: Figure S6 — Biological replicate of transplantation of N202 tumor cell spheroids to brain tissue. This biological replicate was performed exactly as the initial experiment. There is no reason to anticipate that the rate of evolution in two independent experiments would be identical. Therefore, we sought to match the two sequential passages repP1 and repP2 from the replicate experiment to the appropriate position in the original time course, P0-P4. To achieve this, a spline curve was fit to every gene in the plot of passage vs. RMA-normalized gene expression signal. We used these spline curves to fill a matrix of interpolated intermediate values for each gene at intermediate passages, e.g. P0, P0.002, P0.004,…, P4.000. We then calculated the sum of absolute differences between repP1 and every column in this matrix, and assigned repP1 to the column in the matrix where this statistic was minimum. The position assignment was based on 1000 random bootstrap samples of 100 randomly sampled genes. This was repeated for repP2. Histograms indicated that repP1 and repP2 fit best at P2.65 (blue) and P3.82 (red), respectively. The grey bar corresponds to original P2 as an algorithm check. (TIF) [file pone.0101160.s006.tif]

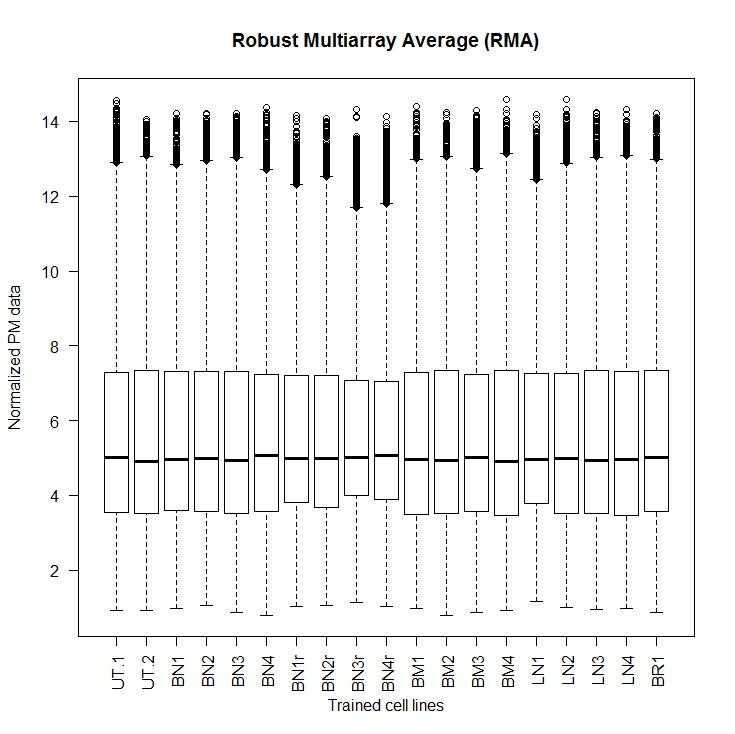

Supplement: Figure S7 — Box plot showing the results of robust multiarray averaging (RMA). The robust multiarray average implemented in Affymetrix Expression Array Console Software. UT = Untrained, BN = brain, BNr = brain technical repeat, BM = bone marrow, LN = lung, BR = breast. Boxes are bounded at the first and third quartiles. Wiskers are at 1.5 times the range. (TIF) [file pone.0101160.s007.tif]

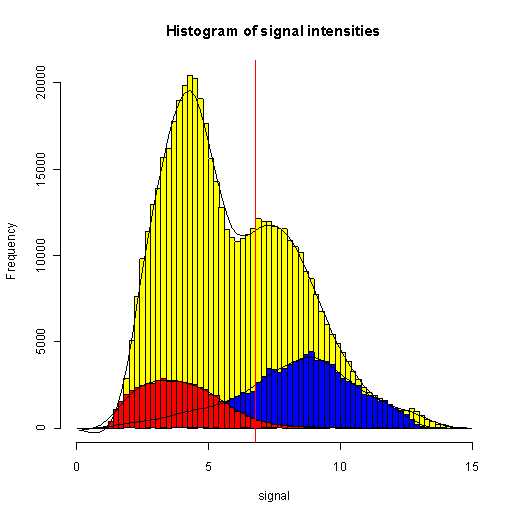

Supplement: Figure S8 — Histogram of PM signals on Affymetrix arrays. Yellow = all PM (perfect match) scores, Red = negative hybridization controls, Blue = positive hybridization “spike-in” controls provided by the manufacturer. The vertical red line is placed at the position below which are 95% of the negative controls. Genes were included for which the signal exceeded this threshold for at least one trained or untrained cell line. (TIF) [file pone.0101160.s008.tif]

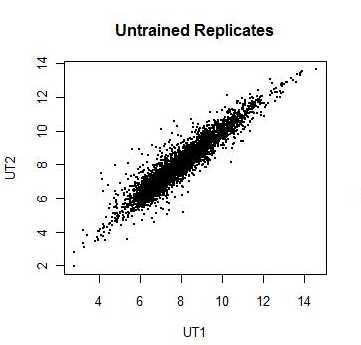

Supplement: Figure S9 — Scatterplot of RMA-normalized untrained control replicates. UT1 and UT2 microarrays were repeated beginning with two independent cell cultures and corresponding RNA preparations, and plotted as a scatterplot. This plot provides a visual demonstration of reproducibility. (TIF) [file pone.0101160.s009.tif]

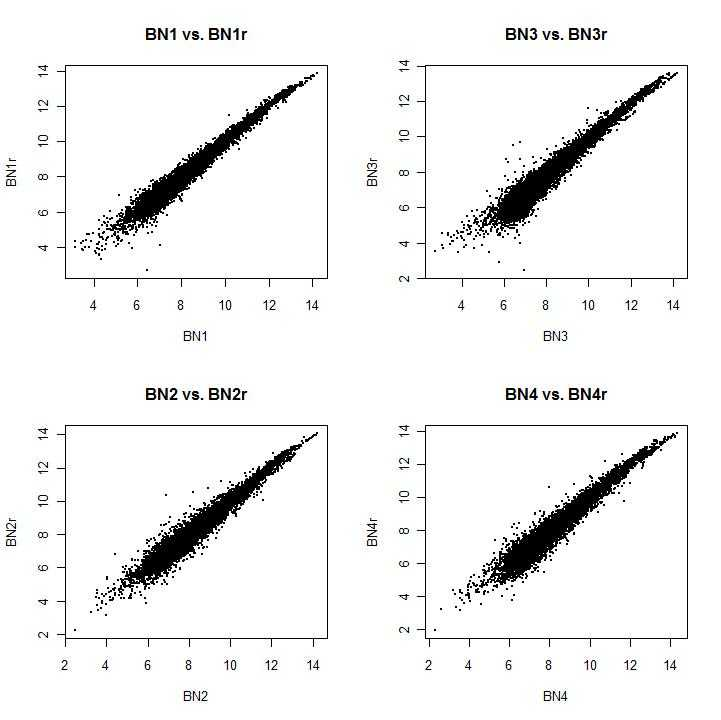

Supplement: Figure S10 — Technical replicate reproducibility. Technical replicates of microarrays for cells trained to grow on brain tissue, beginning with independent cell cultures and corresponding RNA preparations. (TIF) [file pone.0101160.s010.tif]
