# Supplementary material for: Effects of Different Tissue Microenvironments on Gene Expression in Breast Cancer Cells
Source: PLoS One. 2014 Jul 8;9(7):e101160. doi: 10.1371/journal.pone.0101160 (PMC4086928; doi:10.1371/journal.pone.0101160)
Supplement: Table S1 — Significant GO Biological Process Terms related to “transport” associated with genes that are UP-REGULATED upon initial contact with Brain, Bone Marrow, and Lung Tissues. (DOCX) [file pone.0101160.s011.docx]

| **Table S1. Significant GO Biological Process Terms related to “transport” associated with genes that are UP-REGULATED upon initial contact with Brain, Bone Marrow, and Lung Tissues** | | | | |
| --- | --- | --- | --- | --- |
| **GO Number** | **GO Biological Process Term** | **q-value** | **KMG** | **Gene** |
| BRAIN | | | | |
| GO:0051929 | positive regulation of calcium ion transport via voltage-gated calcium channel activity | 0.0491 | 2 | Vdr |
| GO:0032376 | positive regulation of cholesterol transport | 0.0491 | 2 | Lipg |
| GO:0010828 | positive regulation of glucose transport | 0.0491 | 2 | Ak1 |
| GO:0006860 | extracellular amino acid transport | 0.033 | 2 | Slc1a5 |
| GO:0042892 | chloramphenicol transport | 0.033 | 2 | Tlr2 |
| GO:0042953 | lipoprotein transport | 0.0491 | 2 | Apobec1 |
| GO:2000651 | positive regulation of sodium ion transmembrane transporter activity | 3.17E-002 | 6 | Wnk2 |
| GO:0010523 | negative regulation of calcium ion transport into cytosol | 3.17E-002 | 6 | Bcl2 |
| GO:0022898 | regulation of transmembrane transporter activity | 3.17E-002 | 6 | Bcl2 |
| GO:0044341 | sodium-dependent phosphate transport | 3.17E-002 | 6 | Slc34a2 |
| GO:0003333 | amino acid transmembrane transport | 5.55E-003 | 6 | Slc38a1 Slc1a1 Slc7a5 |
| GO:0015807 | L-amino acid transport | 7.83E-004 | 6 | Slc38a1 Slc1a1 Slc7a5 |
| GO:0015798 | myo-inositol transport | 4.72E-002 | 6 | Slc5a3 |
| GO:0006865 | amino acid transport | 2.94E-002 | 6 | Slc38a1 Slc7a5 |
| GO:0006814 | sodium ion transport | 5.98E-004 | 6 | Atp1b1 Slc38a1 Slc5a3 Slc34a2 |
| GO:0015889 | cobalamin transport | 3.17E-002 | 6 | Tcn2 |
| GO:0006868 | glutamine transport | 3.17E-002 | 6 | Slc38a1 |
| GO:0006811 | ion transport | 9.36E-004 | 6 | Atp1b1 Tcn2 Chrnb1 Slc38a1 Slc5a3 Lrrc26 Slc34a2 Atp6v0e2 |
| GO:0015810 | aspartate transport | 4.97E-002 | 8 | Slc25a12 Slc25a13 |
| GO:0006913 | nucleocytoplasmic transport | 1.92E-002 | 11 | Mybbp1a Nup155 Aaas |
| GO:0055085 | transmembrane transport | 1.71E-002 | 11 | Gja1 Slc25a48 Slc4a7 Slc25a37 Nup155 Tomm22 Abcc1 Atp13a3 Slc39a13 Slc23a2 Zmat3 Pom121 Aqp8 Hnrnpa3 |
| GO:0051926 | negative regulation of calcium ion transport | 3.10E-003 | 12 | Ptgs2 |
| GO:0071421 | manganese ion transmembrane transport | 3.03E-002 | 13 | Mmgt2 Slc11a2 |
| GO:0035444 | nickel cation transmembrane transport | 3.03E-002 | 13 | Mmgt2 Slc11a2 |
| GO:0008090 | retrograde axon cargo transport | 4.80E-002 | 13 | Dst Ndel1 |
| GO:0006828 | manganese ion transport | 3.03E-002 | 13 | Mmgt2 Slc11a2 |
| GO:0015675 | nickel cation transport | 3.03E-002 | 13 | Mmgt2 Slc11a2 |
| GO:0034755 | iron ion transmembrane transport | 3.42E-002 | 14 | Scara5 Trf |
| GO:0015991 | ATP hydrolysis coupled proton transport | 4.13E-003 | 15 | Atp6v1f Atp6v0a4 Atp6v1b2 |
| GO:0015986 | ATP synthesis coupled proton transport | 1.25E-002 | 15 | Atp6v1f Atp6v0a4 Atp6v1b2 |
| GO:0010827 | regulation of glucose transport | 4.44E-002 | 15 | Trib3 |
| GO:0006835 | dicarboxylic acid transport | 9.76E-003 | 15 | Slc1a4 Slc1a2 |
| GO:0034589 | hydroxyproline transport | 4.44E-002 | 15 | Slc1a4 |
| GO:0015826 | threonine transport | 4.44E-002 | 15 | Slc1a4 |
| GO:0006820 | anion transport | 3.32E-002 | 15 | Slc1a4 Slc1a2 |
| GO:0015893 | drug transport | 4.44E-002 | 15 | Slc18a1 |
| GO:0006810 | transport | 4.44E-002 | 15 | Rabif Slc5a8 G630090E17Rik Slc29a3 Aldh1l2 Slc1a4 Cacnb3 Slc39a4 Slc25a23 Chmp1b Slc1a2 Slc7a11 Atp6v1f Atp6v0a4 Myo7a Atp6v1b2 Slc18a1 Slc38a7 Scamp5 |
| GO:0035434 | copper ion transmembrane transport | 4.96E-002 | 16 | Slc31a2 Slc31a1 |
| GO:0051928 | positive regulation of calcium ion transport | 2.15E-002 | 17 | Ccl5 |
| GO:0034220 | ion transmembrane transport | 4.39E-003 | 17 | Gabrp Trpm3 Atp6v1b1 |
| GO:0006816 | calcium ion transport | 3.51E-003 | 17 | Ramp1 Ccl5 |
| GO:0006893 | Golgi to plasma membrane transport | 2.14E-002 | 18 | Steap2 |
| GO:0015822 | ornithine transport | 2.14E-002 | 18 | Slc7a2 |
| GO:0015809 | arginine transport | 4.24E-002 | 18 | Slc7a2 |
| GO:0006826 | iron ion transport | 9.55E-003 | 18 | Steap2 Steap1 |
| GO:0015819 | lysine transport | 2.14E-002 | 18 | Slc7a2 |
| GO:0051223 | regulation of protein transport | 1.79E-002 | 19 | Fndc1 |
| GO:0015920 | lipopolysaccharide transport | 1.79E-002 | 19 | Lbp |
| GO:0006836 | neurotransmitter transport | 4.71E-002 | 19 | Slc6a14 |
| GO:0008088 | axon cargo transport | 2.38E-002 | 19 | Ugt8a |
| GO:0015837 | amine transport | 1.79E-002 | 19 | Slc6a14 |
| GO:0032387 | negative regulation of intracellular transport | 4.05E-002 | 22 | Mtap1b |
| GO:0035725 | sodium ion transmembrane transport | 4.05E-002 | 22 | Slc4a11 |
| GO:0035445 | borate transmembrane transport | 4.05E-002 | 22 | Slc4a11 |
| GO:0032600 | chemokine receptor transport out of membrane raft | 4.05E-002 | 22 | Cd24a |
| GO:0042045 | epithelial fluid transport | 4.05E-002 | 22 | Edn1 |
| GO:0032597 | B cell receptor transport into membrane raft | 4.05E-002 | 22 | Cd24a |
| GO:0047497 | mitochondrion transport along microtubule | 4.05E-002 | 22 | Mtap1b |
| GO:0030185 | nitric oxide transport | 4.05E-002 | 22 | Edn1 |
| GO:0015881 | creatine transport | 4.05E-002 | 22 | Slc6a8 |
| GO:0046713 | borate transport | 4.05E-002 | 22 | Slc4a11 |
| GO:0042044 | fluid transport | 4.05E-002 | 22 | Slc4a11 |
| GO:0070588 | calcium ion transmembrane transport | 2.88E-002 | 25 | Atp2b4 Trpv6 |
| GO:0046968 | peptide antigen transport | 4.12E-002 | 25 | Tap2 |
| GO:0046967 | cytosol to ER transport | 2.51E-003 | 25 | Tap1 Tap2 |
| GO:0019060 | intracellular transport of viral proteins in host cell | 2.51E-003 | 25 | Tap1 Tap2 |
| GO:0032218 | riboflavin transport | 4.12E-002 | 25 | 2310046K01Rik |
| GO:0015833 | peptide transport | 4.12E-003 | 25 | Tap1 Tap2 |
| Bone Marrow | | | | |
| GO:0009914 | hormone transport | 0.0279 | 1 | Slc16a2 |
| GO:0006868 | glutamine transport | 2.79E-002 | 1 | Slc38a1 |
| GO:0042892 | chloramphenicol transport | 2.79E-002 | 1 | Tlr2 |
| GO:0055085 | transmembrane transport | 3.40E-002 | 2 | Gja1 Slc19a1 Slc4a7 Vdac2 Slc7a7 Xpo7 Tomm22 Slc25a32 Slc16a1 Slc25a24 Slc7a11 Hnrnpa3 |
| GO:0006820 | anion transport | 7.40E-003 | 4 | Slc4a11 Slc1a5 |
| GO:0042044 | fluid transport | 2.03E-002 | 4 | Slc4a11 |
| GO:0006833 | water transport | 3.02E-002 | 4 | Aqp8 |
| GO:0046713 | borate transport | 2.03E-002 | 4 | Slc4a11 |
| GO:0015825 | L-serine transport | 4.01E-002 | 4 | Slc1a5 |
| GO:0015701 | bicarbonate transport | 3.02E-002 | 4 | Slc4a11 |
| GO:0030185 | nitric oxide transport | 2.03E-002 | 4 | Edn1 |
| GO:0015732 | prostaglandin transport | 3.02E-002 | 4 | Slco2a1 |
| GO:0042045 | epithelial fluid transport | 2.03E-002 | 4 | Edn1 |
| GO:0015804 | neutral amino acid transport | 4.99E-002 | 4 | Slc1a5 |
| GO:0043691 | reverse cholesterol transport | 4.01E-002 | 4 | Lipg |
| GO:0035445 | borate transmembrane transport | 2.03E-002 | 4 | Slc4a11 |
| GO:0015722 | canalicular bile acid transport | 2.03E-002 | 4 | Aqp8 |
| GO:0006860 | extracellular amino acid transport | 2.03E-002 | 4 | Slc1a5 |
| GO:0035725 | sodium ion transmembrane transport | 2.03E-002 | 4 | Slc4a11 |
| GO:0032376 | positive regulation of cholesterol transport | 3.02E-002 | 4 | Lipg |
| GO:0043268 | positive regulation of potassium ion transport | 4.01E-002 | 4 | Cxcl1 |
| GO:0015810 | aspartate transport | 1.04E-002 | 5 | Slc25a12 Slc25a13 |
| GO:0051029 | rRNA transport | 2.42E-002 | 6 | Tst |
| GO:0030050 | vesicle transport along actin filament | 2.42E-002 | 6 | Wasl |
| GO:0022900 | electron transport chain | 1.61E-002 | 6 | Ndufs4 Ero1l Steap1 |
| GO:0015920 | lipopolysaccharide transport | 3.61E-002 | 6 | Lbp |
| GO:0072321 | chaperone-mediated protein transport | 4.79E-002 | 6 | Timm9 |
| GO:0051926 | negative regulation of calcium ion transport | 3.61E-002 | 6 | Ptgs2 |
| GO:0006829 | zinc ion transport | 4.69E-002 | 24 | Slc39a10 Slc39a14 Slc39a13 |
| GO:0006810 | transport | 4.41E-002 | 25 | Slc5a8 Slc39a4 Slc26a2 Fads1 Slc1a1 Sorbs1 Slc2a6 Sort1 Slc5a9 Atp6v0e2 Atp6v0a4 Gga2 Trf |
| GO:0032509 | endosome transport via multivesicular body sorting pathway | 2.79E-002 | 25 | Sort1 |
| GO:0015682 | ferric iron transport | 2.79E-002 | 25 | Trf |
| GO:0048227 | plasma membrane to endosome transport | 2.79E-002 | 25 | Sort1 |
| GO:0015986 | ATP synthesis coupled proton transport | 3.44E-002 | 25 | Atp6v0e2 Atp6v0a4 |
| GO:0015991 | ATP hydrolysis coupled proton transport | 1.59E-002 | 25 | Atp6v0e2 Atp6v0a4 |
| GO:2000651 | positive regulation of sodium ion transmembrane transporter activity | 2.79E-002 | 25 | Wnk2 |
| LUNG | | | | |
| GO:0006886 | intracellular protein transport | 6.88E-003 | 5 | Tgfbrap1 Ap1s3 Ap3m1 Hgs Rffl Gga3 Bcap29 Snx6 Xpo7 Clta Ipo13 |
| GO:0006888 | ER to Golgi vesicle-mediated transport | 3.95E-002 | 6 | Cul3 Sec23b Vcp Htt |
| GO:0042953 | lipoprotein transport | 9.16E-003 | 8 | Apobec1 |
| GO:0006826 | iron ion transport | 3.91E-002 | 8 | Steap3 |
| GO:0055085 | transmembrane transport | 1.01E-002 | 12 | Mfsd6 Slc25a35 Slc1a4 Slc35b3 Slc4a7 Slc15a2 Atp8b1 Mfsd2a Clcn6 Slc34a2 Slc24a6 Slc25a38 Slc17a5 Slc6a14 |
| GO:0006814 | sodium ion transport | 1.23E-002 | 12 | Atp1b1 Slc4a7 Slc34a2 Slc24a6 |
| GO:0048227 | plasma membrane to endosome transport | 1.72E-002 | 16 | Sort1 |
| GO:0003333 | amino acid transmembrane transport | 1.51E-002 | 16 | Slc38a1 Slc1a1 |
| GO:0006895 | Golgi to endosome transport | 3.41E-002 | 16 | Sort1 |
| GO:0015807 | L-amino acid transport | 3.82E-003 | 16 | Slc38a1 Slc1a1 |
| GO:0006868 | glutamine transport | 1.72E-002 | 16 | Slc38a1 |
| GO:0032509 | endosome transport via multivesicular body sorting pathway | 1.72E-002 | 16 | Sort1 |
| GO:0001579 | medium-chain fatty acid transport | 4.49E-002 | 20 | Slc27a1 |
| GO:0032218 | riboflavin transport | 4.49E-002 | 20 | 2310046K01Rik |
| GO:0032376 | positive regulation of cholesterol transport | 3.26E-002 | 23 | Lipg |
| GO:0043691 | reverse cholesterol transport | 4.32E-002 | 23 | Lipg |
| GO:0042892 | chloramphenicol transport | 2.18E-002 | 23 | Tlr2 |
| GO:0006811 | ion transport | 2.44E-003 | 23 | Slc5a8 Slc39a4 Slc31a2 Cachd1 Steap4 Atp6v0a4 |
| GO:0051926 | negative regulation of calcium ion transport | 1.88E-002 | 24 | Ptgs2 Inpp5k |
| GO:0046967 | cytosol to ER transport | 1.88E-002 | 24 | Tap1 Tap2 |
| GO:0019060 | intracellular transport of viral proteins in host cell | 1.88E-002 | 24 | Tap1 Tap2 |
| GO:0016197 | endosomal transport | 2.92E-002 | 24 | Vps4b Cdc42 Mcoln1 |
| GO:0015833 | peptide transport | 3.02E-002 | 24 | Tap1 Tap2 |
