# Supplementary material for: Effects of Different Tissue Microenvironments on Gene Expression in Breast Cancer Cells
Source: PLoS One. 2014 Jul 8;9(7):e101160. doi: 10.1371/journal.pone.0101160 (PMC4086928; doi:10.1371/journal.pone.0101160)
Supplement: Table S2 — Genes associated with GO Biological Process terms discussed in the text. (DOCX) [file pone.0101160.s012.docx]

| **Table S2. Genes associated with GO Biological Process terms discussed in the text.** | | | | |
| --- | --- | --- | --- | --- |
| **KMG** | **GO number(*)** | **GO Biological Process Term** | **q-value**** | **Genes** |
| Brain | | | | |
| BN_25_ 2 | GO:0032321(1) | *positive regulation of Rho GTPase activity* | 0.001 | Cdc42ep2, Jun, Grhl3 |
|  | GO:0030036(1) | *actin cytoskeletal organization* | 0.003 | Coro6, Pdgfb, Cdc42ep2, Phactr4, Fscn1 |
|  | GO:0032956(1) | *regulation of actin cytoskeleton organization* | 0.016 | Grhl3, Palld |
|  | GO:0051017(1) | *actin filament bundle assembly* | 0.019 | Actn1, Fscn1 |
| BN_25_ 4 | GO:0007049(1) | *cell cycle* | 7x10^-11^ | Terf1, Sgol2, Aspm, Nuf2, Fbxo5, E2f7, Aurkb, Cdc6, E2f6, Vrk1, Ncapg2, C79407, Cinp, Cks2, Dlgap5, F630043A04Rik, Esco2, Cdca2, Racgap1, Cdc45, Mapre2, Cdca5, Kif20b, Kif11, Cep55, Incenp, Anxa1, Fam83d, Spc25, Bub1, Smc4, Psrc1, Sass6, Ect2, Ccna2, Ccne2, Casp8ap2, Smc2, Nasp, Kif2c, Nudc, Kntc1, Stard13, Mad2l1, Cdca3, Ncapd2, Fanci, E2f8, Spc24, Ccnb2, Cetn2 |
|  | GO:0051301(1) | *cell division* | 4x10^-7^ | Terf1, Sgol2, Aspm, Nuf2, Fbxo5, Aurkb, Cdc6, Vrk1, Ncapg2, C79407, Cinp, Cks2, F630043A04Rik, Cdca2, Racgap1, Cdc45, Mapre2, Cdca5, Kif20b, Kif11, Cep55, Incenp, Fam83d, Spc25, Bub1, Smc4, Psrc1, Ect2, Ccna2, Ccne2, Smc2, Kif2c, Nudc, Kntc1, Mad2l1, Cdca3, Ncapd2, Spc24, Ccnb2, Cetn2 |
|  | GO:0007067(1) | *mitosis* | 2x10^-6^ | Terf1, Aspm, Nuf2, Fbxo5, Aurkb, Cdc6, Vrk1, Ncapg2, C79407, F630043A04Rik, Cdca2, Mapre2, Cdca5, Kif20b, Kif11, Cep55, Incenp, Fam83d, Spc25, Bub1, Smc4, Psrc1, Ccna2, Smc2, Kif2c, Nudc, Kntc1, Mad2l1, Cdca3, Ncapd2, Spc24, Ccnb2, Cetn2 |
|  | GO:0006260(1) | *DNA replication* | 8x10^-6^ | Dna2, Prim1, Cdc6, Pold2, Tk1, Rrm2, Pole2, Cinp, Cdc45, Pola2, Fen1, Mcm10, Orc1, Orc3, Nasp, Rfc5, Rpa3 |
|  | GO:0007076(1) | *mitotic chromosome condensation* | 4x10^-7^ | Cdca5, Smc4, Smc2, Ncapg, Ncapd2 |
|  | GO:0006281(1) | *DNA repair* | 2x10^-4^ | Exo1, Brip1, Tdp1, Gen1, Cinp, Parp2, Esco2, Fen1, Smc4, Rad54b, Smc2, Usp1, Rad54l, Gtf2h3, Rpa3, Mbd4, Fanci, Neil3, Fancb, Cetn2 |
|  | GO:0030261(1) | chromosome condensation | 4.14x10^-4^ | Top2a, Ncapg2, Smc4, Smc2, Ncapd2 |
|  | GO:0007059(1) | chromosome segregation | 9.66x10^-4^ | Sgol2, Top2a, F630043A04Rik, Incenp, Spc25, Bub1, Ebna1bp2, Cdk5rap2, Kif2c |
|  | GO:0006334(1) | nucleosome assembly | 0.001 | Hist1h2bb, Hist1h1b, Hist1h2bh, Hist2h2ab, Hist2h2ac, Cenpa, Asf1b |
|  | GO:0006487(1) | protein N-linked glycosylation | 0.002 | Entpd5, Alg6, Pomgnt1, Ddost |
|  | GO:0006284(1) | base-excision repair | 0.006 | Dna2, Parp2, Mbd4, Neil3 |
|  | GO:0007099(1) | centriole replication | 0.009 | Plk4, Sass6, Cetn2 |
| BN_25_ 15 | GO:0001569(2) | *patterning of blood vessels* | 0.004 | Plxnd1, Vangl2, Vegfa |
|  | GO:0001944(2) | *vascular development* | 0.004 | Fzd5, Fzd2, Vegfa |
|  | GO:0034976(3) | *response to endoplasmic reticulum stress* | 0.001 | Fam129a, Ddit3, Scamp5 |
|  | GO:0030968(3) | *endoplasmic reticulum unfolded protein response* | 0.006 | Ddit3, Ppp1r15a, Herpud1 |
| BN_25_ 18 | GO:0055072 | *iron ion homeostasis* | 0.0009 | Steap2, Steap1, Hmox1 |
|  | GO:0015809 | *arginine transport* | 0.042 | Slc7a2 |
| BN_25_ 19 | GO:0015837 | *amine transport* | 0.018 | Slc6a14 |
|  | GO:0008228(3) | *opsonization* | 0.0003 | Sftpd, Lbp |
|  | GO:0006935(3) | *chemotaxis* | 0.0004 | S100a8, Kit, Cxcl5 |
|  | GO:0043032(3) | *positive regulation of macrophage activation* | 0.0007 | Il33, Lbp |
|  | GO:00507866(3) | *positive regulation of phagocytosis* | 0.002 | Sftpd, Sirpa |
| BN_25_ 20 | GO:0001525(2) | *angiogenesis* | 0.003 | Plau, Col8a1, Nrp1 |
| BN_25_ 25 | GO:0015833(3) | *peptide transport* | 0.008 | Tap1, Tap2 |
|  | GO:0006508(3) | *proteolysis* | 0.01 | Lgmn, Adamts1, Adamts5, Psmb9, Eml3, Ctsw, Ermp1, Ctso, Ece1 |
|  | GO:0045087(3) | *innate immune response* | 0.02 | Jak2, Ifih1, Zbp1, Sp110 |
| Bone | | | | |
| BM_25_ 4 | GO:0045670(4) | *regulation of osteoclast differentiation* | 0.0006 | 5730469M10Rik, Adam8 |
| BM_25_ 19 | GO:0030500(4) | *regulation of bone mineralization* | 0.0004 | Ank, Ecm1, Mgp |
|  | GO:0001525(4) | *angiogenesis* | 0.0006 | Elk3, Plau, Hbegf, Ccbe1, Ecm1, Epha2 |
| Lung | | | | |
| LN_25_ 19 | GO:0060484(5) | *lung-associated mesenchyme development* | 0.001 | Ptk7, Hoxa5, Fgfr1 |
|  |  |  |  |  |

*Category coding: (1) Cell cycle, adhesion, and motility (maroon), (2) Angiogenesis (red) , (3) Endoplasmic Reticulum Stress and Innate Immunity (blue), (4) Bone marrow (gray), (5)Various (black).

**The q-values correspond to p-values corrected for multiple testing, as described in the text.
