# Supplementary material for: Effects of Different Tissue Microenvironments on Gene Expression in Breast Cancer Cells
Source: PLoS One. 2014 Jul 8;9(7):e101160. doi: 10.1371/journal.pone.0101160 (PMC4086928; doi:10.1371/journal.pone.0101160)
Supplement: Supplementary file 4 [file pone.0101160.s013.docx]

Table S3. Genes strongly induced or repressed in different passages in brain tissue.

Timing	Expression Behavior	Gene
highest in P1	≥4-fold up-regulated	Akr1b3, Ccl9, Crct1, Gm6644, Jam2, Kctd12b, Klk6, Klk9, Lrrk2, Maoa, Mcpt8, Moxd1, Neto2, Nr4a2, Nt5dc2, Pcdh7, Pgap1, Pilrb2, Rnasel, Slc1a1, Slc7a2, Slit2, Sparc, Steap1
highest in P1 and P2	≥4-fold up-regulated, approximately equal in P1 and P2	Atp1b1, Car6, Chi3l1, Chrm3, Cldn1, Cxcl5, Fam73a, Gpx2, Hlf, Il1f6, Isyna1, Itga1, Kit, Lass6, Ltf, Man1c1, Pilrb1, Prss12, S100a8, Sftpd, Sirpa, Slc38a1, Slc6a14, Syne1, Tmem47, Tnfrsf21, Ugt8a, Zc4h2.
highest in P2	≥4-fold up-regulated	Akap12, Aldh1l2, Armcx2, Chac1, Chrm3, Csn3, Ctxn1, Cxx1a, Dbn1, Ddit3, Dip2c, Ehf, Fam129a, Fndc1, Gadd45a, Gdpd1, Gjb3, Gm129, Gpt2, Gpx2, Gvin1, Il33, Klhl38, Klk14, Lbp, Mlana, Mthfd2, Phyhd1, Pigr, Plekhb1, Reep6, Renbp, Tc2n, Tmem150a, Trib3, Wif1.
highest in P3	≥4-fold up-regulated	2310002L13Rik, Aicda, Akr1b8, Anxa8, Gsta1, Has2 , Lce1h, Lce1i, Ly6c1, , Ly6d, Rbp7, Serpine1
highest in P3 and P4	≥4-fold up-regulated, approximately equal in P3 and P4	Lce1g, Ly6a, Ly6c2
highest in P4	≥4-fold up-regulated	Aicda, Car9, Ddr2, Il2rg, Ly6a, Ly6c2, Olfml3, Orm1
high in P4 in all tissues	≥2-fold up-regulated	2310002L13Rik, Aicda, Akr1b8, Anxa8, Arhgdib, Cbr2, Cxcl5, Gsta1, Has2, Il1f6, Il2rg, Lass3, Ly6a, Ly6c1, Ly6c2, Ly6d, Mela, Olfml3, Pcolce, Pcolce2, Ppbp, Prnd, Rbp7, Reep6, Rnd1, S100a8, Samd12, Serpine1, Tnip3, U90926
down in P4	~4-fold down-regulated in P4	1300014I06Rik, 1500015010Rik, Adamts9, Atp10b, BC005685, Ccdc141, Clca5, Cldn2, Cpm, Epha4, Eya1, Eya2, Gadrp, Gstm1, Hey1, Lrt23, Lect1, Ms4a6d, Ppargc1a, Sd[r, Sema7a, Slc38a1, Slco1a5,St8sia1, Trpm3
higher than control in all tissues in at least one passage	≥2-fold up-regulated in all tissues in at least one passage	2310002L13Rik, 9930023K05Rik, Adam8, Afap1l1, Aicda, Akr1b8, Alpl, Anxa8, Apobec1, Arhgdib, Ass1, Atp10d, Atp6v0a4, C630004H02Rik, Car9, Cbr2, Cdc42ep2, Cdhr1, Cldn1, Csf3, Cxcl1, Cxcl5, Dse, Edn1, Fosl1, Fxyd5, Gatsl3, Gm10639, Gsta1, Gsta2, Has2, Il1f6, Il2rg, Itgb6, Lass3, Lipg, Ltf, Ly6a, Ly6c1, Ly6c2, Ly6d, Ly96, Lypd3, Mela, Muc20, Ndrg1, Olfml3, Pak3, Palld, Pcolce, Pcolce2, Pdgfb, Pilrb2, Plscr2, Ppbp, Prkch, Prnd, Ptgs2, Rbp7, Reep6, Rgs16, Rnasel, Rnd1, S100a14, S100a8, Samd12, Serpine1, Sh3bp1, Slco2a1, Stc2, Tigit, Timp1, Tlr2, Tnip3, Tspan1, U90926
lower than control in P4 in all tissues	≥2-fold down-regulated in all tissues in P4	1500015O10Rik, Ak3, Atp10b, Atp6v1b1, BC005685, Ccl5, Cldn2, Epha4, Eya1, Gabrp, Gstm1, Gzme, Hey1, Lect1, Maoa, Mcpt8, Ms4a6d, Plekhb1, Ppargc1a, Sdpr, Sema7a, Slco1a5, Slit2, Trpm3, Zc4h2.
down-regulated in all tissues in at least one passage	≥2-fold down-regulated in all tissues in any passage	1500015O10Rik, Adamts9, Ak3, Aldh3a1, Anxa9, Atp10b, Atp6v1b1, AW112010, BC005685, Cadm1, Car2, Ccl5, Ceacam1, Clca5, Cldn2, D10Bwg1379e, Dll1, Dusp6, Efna3, Epha4, Etv1, Eya1, Eya2, Fn1, Foxi1, Gabrp, Galnt7, Gm14005, Grb14, Gstm1, Gtpbp10, Gzme, Hey1, Hsd11b1, Ifi44, Kcnn4, Krt23, Lama4, Lbh, Lect1, Maoa, Mcpt8, Mlst8, Ms4a6d, Papln, Pgf, Pilra, Pla2g16, Plekhb1, Ppargc1a, Ramp1, Rny1, S100a4, Saa3, Sdpr, Sema5a, Sema7a, Sftpd, Slc1a1, Slco1a5, Slit2, Snora44, St8sia1, St8sia6, Tgfbr3, Timp3, Trpm3, Tspan13, Txnip, Unc80, Zc4h2
up in P4	strongly up-regulated (≥4-fold) in all three tissues by P4	Aicda, Akr1b8, Anxa8, Has2, Il2rg, Ly6a, Ly6d, Orm1, Serpine1 and Rpb7
